# The *Giardia lamblia vsp *gene repertoire: characteristics, genomic organization, and evolution

**DOI:** 10.1186/1471-2164-11-424

**Published:** 2010-07-09

**Authors:** Rodney D Adam, Anuranjini Nigam, Vishwas Seshadri, Craig A Martens, Gregory A Farneth, Hilary G Morrison, Theodore E Nash, Stephen F Porcella, Rima Patel

**Affiliations:** 1 Departments of Medicine and Immunobiology, University of Arizona College of Medicine, Tucson, AZ, USA; 2 Dept of Immunobiology, University of Arizona College of Medicine, Tucson, AZ, USA; 3 Genomics Unit, Research Technologies Section, RTB, NIAID, NIH, Rocky Mountain Laboratories, Hamilton, MT, USA; 4 Josephine Bay Paul Center, MBL, Woods Hole, MA, USA; 5 Laboratory of Parasitic Diseases, NIAID, National Institutes of Health, Bethesda, MD, USA; 6 Dept of Immunobiology, University of Arizona College of Medicine, Tucson, AZ, USA

## Abstract

**Background:**

*Giardia lamblia *trophozoites colonize the intestines of susceptible mammals and cause diarrhea, which can be prolonged despite an intestinal immune response. The variable expression of the variant-specific surface protein (VSP) genes may contribute to this prolonged infection. Only one is expressed at a time, and switching expression from one gene to another occurs by an epigenetic mechanism.

**Results:**

The WB *Giardia *isolate has been sequenced at 10× coverage and assembled into 306 contigs as large as 870 kb in size. We have used this assembly to evaluate the genomic organization and evolution of the *vsp *repertoire. We have identified 228 complete and 75 partial *vsp *gene sequences for an estimated repertoire of 270 to 303, making up about 4% of the genome. The *vsp *gene diversity includes 30 genes containing tandem repeats, and 14 *vsp *pairs of identical genes present in either head to head or tail to tail configurations (designated as inverted pairs), where the two genes are separated by 2 to 4 kb of non-coding DNA. Interestingly, over half the total *vsp *repertoire is present in the form of linear gene arrays that can contain up to 10 *vsp *gene members. Lastly, evidence for recombination within and across minor clades of *vsp *genes is provided.

**Conclusions:**

The data we present here is the first comprehensive analysis of the *vsp *gene family from the Genotype A1 WB isolate with an emphasis on *vsp *characterization, function, evolution and contributions to pathogenesis of this important pathogen.

## Background

*Giardia lamblia *(syn. G. duodenalis, G. intestinalis) is an anaerobic protist that is medically important as a common cause of intestinal infection and diarrhea [1]. Humans and other susceptible mammals become infected when cysts are ingested from contaminated water or food and excyst into trophozoites in the proximal small intestine. These trophozoites replicate and cause the symptoms of diarrhea. Infections with *Giardia *are frequently prolonged and malabsorption with weight loss may last for months in the absence of treatment, despite an immune response that would be expected to eradicate the infection. One of the possible reasons for the persistence of infection is antigenic variation of the variant-specific surface proteins (VSPs).

A single trophozoite expresses only a single member of this protein family at any one time [2], but may switch expression from one VSP to another *in vitro *at a rate which has been estimated at once every 6 to 13 generations [3]. Antigenic variation has also been identified in gerbils [4], mice [5,6], and humans [7], and occurs during the process of encystation/excystation [8,9]. Antigenic variation occurs in the absence of alteration of DNA sequence or chromosomal location of the *vsp *genes [10-12], and likely occurs by epigenetic mechanisms involving histone acetylation status [12] and/or RNAi [13].

The VSP proteins are all cysteine-rich and have frequent CXXC motifs [1]. They have a 14 to 17 amino acid N-terminal signal peptide which is predicted to direct the protein to the trophozoite surface, upon which it is cleaved from the remainder of the peptide [14,15]. Most of the mature VSP protein diffusely coats the outside of the trophozoite [16].

The C-terminus concludes with a nearly invariant CRGKA motif that most likely remains in the cytoplasm [17]. Immediately adjacent to the CRGKA motif is a hydrophobic domain that likely forms the membrane anchor for the VSP [17,18]. The entire C-terminal conserved region is about 38 amino acids in length, but the upstream portions of the VSPs can be highly different, allowing the organism to expose very different antigens to the host.

Despite the similarities in the N-terminal signal peptide, the CXXC motifs, and the conserved C-terminus, other features of the *vsp *genes vary substantially, such as the presence or absence of tandem repeats. *Vsp*A6 is about 5.6 kb in length, with the initial 99 bp followed by 23+ copies of a 195 bp tandem repeat, then about 1.3 kb of additional sequence that ends with the highly conserved 3' end [10]. Tandem repeats have also been reported in other *vsp*s [11,19-21]. The *vsp*C5 gene is over 2 kb in length and begins with 66 bp at the 5' end followed by over 20 copies of a 105 bp tandem repeat that extended to the conserved 3' end [11]. Thus, almost the entire portion of this molecule that is assumed to be exposed to the extracellular environment consists of tandem repeats.

One *vsp *gene has been described that is encoded as a duplicated pair. The two copies of the *vsp*1267 ORF exist as an inverted pair in a tail to tail orientation with approximately 3 kb of intervening DNA [17].

Previous studies have shown that *vsp *genes fall into related groups or families. One example is *vsp*A6 and the related genes, *vsp*A6-S1 and A6-S2; all sequenced from the WB isolate [22]. *Vsp*A6-S2 differs from *vsp*A6 by its possession of a 201 bp tandem repeat that is similar to the 195 bp repeat of *vsp*A6. *Vsp*A6-S1 is nearly identical to *vsp*A6 with only scattered substitutions outside the repeat region, but has only a bit more than one copy of the 195 bp tandem repeat, rather than the approximately 23 copies found in *vsp*A6. Interestingly, *vsp*A6-S1 has also been sequenced from a Genotype A1 Peruvian isolate called G3 M [23], and both sequences were identical, suggesting that across Genotype A1 isolates, some *vsp *genes may not be changing rapidly.

An additional example of *vsp *genes from Genotype A1 isolates falling within related families includes several genes that are similar to *TSA417 *[24], including *tsp11 *[25-27], such that homology is apparent throughout the entire sequence. In addition, there are *vsp*s with near identity in the upstream non-coding region followed by substantial divergence within the coding region [28].

A genomic evaluation of the vsp genes has been made possible by the sequencing of the genome of the WB isolate [29] 10× coverage (greater than 98% coverage) [30]. The G. lamblia isolates obtained from humans were originally divided into three groups; 1, 2, and 3. These groups are now designated as Genotypes/assemblages A1, A2, and B, respectively. Genotype A1 is a highly homogeneous group and is about 98-99% identical to Genotype A2. Relatively little sequence data is available for Genotype A2. Genotype B is only about 80-90% identical to Genotype A1 and has been proposed as a separate species [31-33]. Although the genome of the Genotype B isolate, GS, has been reported, the coverage was not sufficient to fully describe the vsp repertoire. Therefore, the current manuscript will focus on the Genotype A1 vsp repertoire from the WB genome, which was completed at 10× coverage and assembled into 306 contigs, the largest of which is nearly 1 Mb in size [30].

## Results and Discussion

### Identification of *vsp *genes

All the *vsp *genes are cysteine-rich with frequent CXXCs motifs present throughout their length [34]. However, the *Giardia *genome also encodes other cysteine-rich proteins including three cyst-wall protein (CWP) genes [35] and 61 High Cysteine Membrane Proteins (HCMPs) [36]. The HCMPs also have CXXC motifs, but in addition, they have frequent CXC motifs, which are distinctly uncommon in the VSPs. Most notably, the *vsp *genes differ from these other genes encoding cysteine-rich proteins by their possession of a highly conserved 3' end that concludes with the translated amino sequence, CRGKA. For *vsp*s that were complete at the 3' end, a great majority contained the encoded CRGKA motif in its entirety, while a few genes matched at three or four residues.

Using these criteria, we identified a total of 303 *vsp *genes in the current WB assembly, of which 228 were complete and 75 were in partial or incomplete ORF format. The 228 complete genes include 10 pairs of identical coding inverted gene pairs, so many of the analyses of complete genes included 218 sequences. (A complete list of the *vsp *genes along with their features is shown in Additional file 1). Of the 75 partial genes that were considered in some of the analyses, 32 were incomplete at the 3' end and 43 were incomplete at the 5' end. Since the genome coverage is >98%, it is unlikely that any *vsp *genes were missed completely, although it is possible that some were excluded from the assembly because of the potential for complete or near 100% sequence identity to other *vsp *genes. The total number of 303 *vsp *genes is probably a high estimate for the total genomic repertoire since it is likely that some of the genes that are incomplete at the 5' end should or could have been paired with genes that are incomplete at the 3' end. Therefore, for the purposes of discussion, we have estimated the total number of *vsp *genes to be 228 + 43 or 271 total. These estimates are consistent with earlier range estimates of 150 to 300 that were based on DNA hybridization studies [3].

The 303 genes have been numbered sequentially, based upon their contig number and location. The contigs were numbered sequentially by decreasing size. Therefore, *vsp *genes whose names include large numbers indicate that these *vsps *are found on smaller contigs. Those *vsp*s that have a genomic context as duplicated, inverted pairs and that therefore have identical coding regions, are each given the same numerical designation, but these names are followed by ".1" or ".2" sub-designation to highlight this difference.

The *vsp *coding regions comprise approximately 3% of the total genome. The inclusion of the upstream intergenic areas, which may be tasked with control of *vsp *gene expression, increased the value to greater than 4%. Thus, the *vsp *genes comprise at least 3-4% of the total *Giardia *genome, which is consistent with estimates from a previous *Giardia *genome sampling survey [37].

### The presence of a candidate Inr

All of the *vsp *genes previously described in the literature have had the following DNA consensus sequence, PyAatgTT, at the beginning of the coding region, where atg represents the initiation codon. We have shown in transient transfection studies that this consensus sequence is required for efficient expression of luciferase from a *vsp *promoter region (unpublished). Therefore, this consensus region surrounding the start codon is a candidate Initiator Element (Inr). We analyzed the *vsp *gene sequences for the presence of the PyAatgTT sequence and found that fewer than half of the genes contained this Inr sequence. In other words, 107, or 41% of the 260 *vsp *genes that were complete at the 5' end contained the PyAatgTT sequence. Specifically, 65 *vsp *genes contained putative Inrs of "CAatgTT", while 42 *vsp *genes contained the sequence "TAatgTT". As described above and below, many of the *vsp *genes were found in linear arrays in the genome. Of the 25 *vsp *genes that represented the first *vsp *gene within the linear arrays, 12 contained the putative Inr. In surprising contrast, none of the downstream members of the linear arrays contained this sequence. In total, 107 (72%) of the 148 complete *vsp *genes that were not present as downstream members of linear arrays contained the conserved Inr sequence. For those *vsp*s containing the putative Inr, the Inr was usually (74%) the first observed or most 5' oriented ATG start codon of the gene. None of the subsequent downstream, in-frame encoded ATGs contained a complete PyAatgTT sequence. Further investigation and analysis will be required to determine the role the Inr plays in *vsp *expression across the multitude of *vsp *variety and whether the presence or absence of the Inr plays a role in *in vivo vsp *expression.

### *vsp *genes without an ATG start codon may represent pseudogenes

Three of the 218 *vsp *genes with complete sequences available had no in frame ATG that could be found. These three genes were identified by their possession of the conserved 3' sequence concluding with an encoded CRGKA and were all found in the middle of large contigs. They were relatively short *vsp*-encoding sequences with base pair encoding regions of 224, 329, and 902 bases following the upstream stop codon. There were no obvious differences between these three genes and those containing an ATG. Because of their internal contig location and the absence of any mis-assembly evidence, we believe these three genes may represent inactive, truncated *vsp *pseudogenes.

### ***Vsp *size range, CXXC motifs, tandem encoded repeats and recombination analysis of *vsp*s**

***Vsp *size range and CXXC motifs **were analyzed within the 218 unique *vsp*s. The data demonstrated a wide range in *vsp *size, from 222 to 6777 base encoding pairs. CXXC motif analysis found an average of 12.6 encoded CXXCs motifs per kb (range 3.6 to 18.6 per kb). The vast range in *vsp *size along with differing numbers of CXXC motifs implies a diverse range of function or performance within the *vsp *family. More work remains to be performed in order to analyze the context of VSP protein structure, function and/or surface expression in the context of *vsp *gene size and the presence of high or low numbers of CXXC motifs.

**Tandem repeats (TR) **are a feature of some VSPs and have been shown to be immunodominant in the case of the 195 bp repeat domain found in *Vsp*A6 [19]. Thus, the number and type of repeats encoded in *vsp*s are likely to play an important role in modulating, directing or avoiding the host immune response.

**Figure 1:**
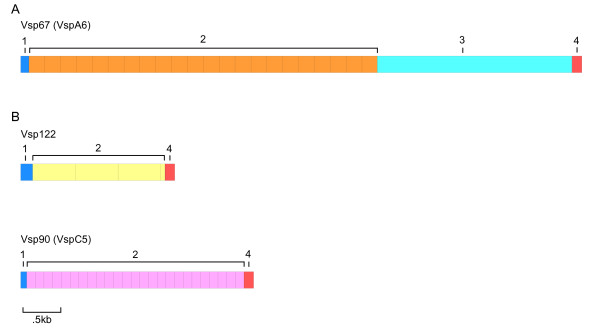
**Examples of *vsp *genes with tandem repeats**. Three examples of *vsp *genes with tandem repeats are shown. The ORFs are divided into 3 or 4 sections, numbered 1 through 4 on the diagrams. For these 3 genes, the first section consists of 66 to 99 bp beginning with the start codon and encoded leader peptide, and extending to the tandem repeats. The second section consists of multiple tandem repeats, which for two of the *vsp *genes (B and C), extends up to the conserved 3' region. The third section (A only) is nonrepetitive DNA extending from the tandem repeats to the conserved 3' region. (1) *Vsp*67 (also CRP170, *vsp*A6) has approximately 22 copies of a 195 bp tandem repeat [10]. (2) *vsp*122 has three copies of a 516 bp repeat, by far the largest repeat found. (3) *vsp*90 (*vsp*C5) has approximately 25 copies of a 105 bp tandem repeat [11].

We identified a total of 30 complete *vsp *genes containing TR (Fig 1). The sequences of the TR were different and unique for each of the 30 genes identified (see the phylogenetic alignment of the tandem repeats in Additional file 2). The nucleotide sequences of the individual repeats ranged in size from 105 to 516 bp in length. The number of copies of the TR ranged from two, to more than 20 per *vsp*. It is important to note that the repeat copy numbers should be viewed as approximate because *vsp *genes with larger numbers of repeats may have been artificially condensed by the genome assembly process. In addition, there is evidence of allelic variability in repeat copy number for at least two of the previously identified repeat-containing *vsp *genes *vsp*A6 and *vsp*C5 [10,11,28].

### *vsp *Conserved 3' domain

A mechanism by which expression status of multiple *vsp*s could be monitored simultaneously would be valuable in the continued study of this interesting gene family. The conserved 3' end has enough similarity among *vsp*s to allow the development of "universal amplification primers". Therefore, we analyzed the conserved 117 nucleotides of the 3' terminus in the 254 *vsp *genes for which this sequence was available to determine whether there was sufficient diversity within the 3' end to allow unique identification of most *vsp*s. Among these 254 genes, there were 190 distinct 3' sequences. These 190 sequences encode 99 different amino acid sequences. Thus, the great majority of *vsp *genes have unique 3' conserved regions. The nucleotide alignments are shown in Additional file 3 and the amino acid alignments are shown in Additional file 4.

### **DNA alignments and evidence for DNA recombination between *vsp *genes**

**Figure 2:**
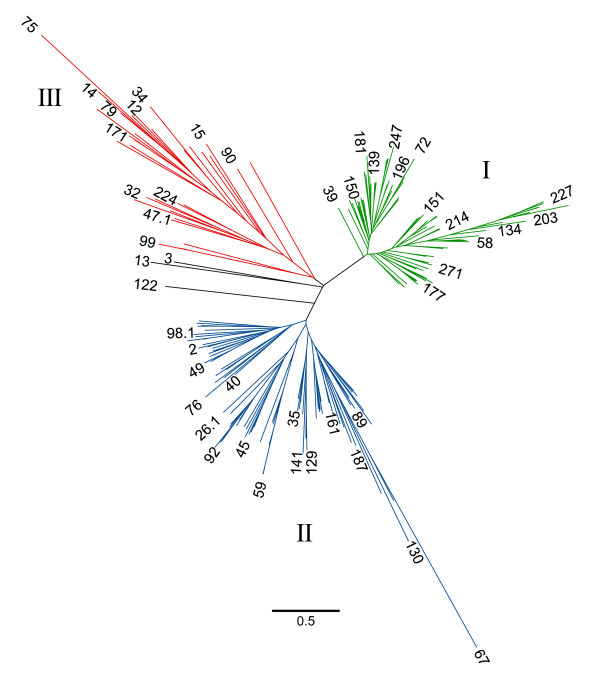
**Unrooted phylogenetic tree of the translated amino acid sequences of all 218 *vsp *genes with complete sequences**. Full length *vsp *proteins were aligned, the alignment manually corrected, and an unrooted tree produced. Predominate clades are listed as I, II, and III and colored Green, Blue and Red. Many of the 218 *vsp *names are not shown on this tree due to confined space at the ends of the branch points. Representative members of each clade are shown. The bar at the bottom of the figure signifies branch length related to number of substitutions per residue analyzed.

It was previously proposed that gene duplication followed by divergence was one of the mechanisms by which the current number and diversity of the *vsp *repertoire was generated [22]. The sequence diversity of the DNA sequences is illustrated by the phylogenetic tree shown in Additional file 5). Since prior studies suggested recombination among vsps [28], we performed a systematic search for evidence of horizontal movement of DNA sequences in the form of recombination between *vsp *genes. Recombination analysis of all 218 full length *vsp*s was not possible due to the wide range of sequence divergence across the entire gene family (data not shown). However, a more focused approach by (1) utilizing the C-terminus conserved region as an alignment reference point for all *vsp*s, and (2) gradually adding additional, longer upstream sequences allowed us to identify minor clades of *vsp*s for running alignments, generating trees, and testing for evidence of recombination. We used the Sawyers test in the Geneconv package of algorithms for these minor clades analyses. For each incremental increase of adjacent upstream 3' sequence, alignments and trees produced greater diversity and longer branching patterns, which correlated with the increased diversity seen in the 5' coding regions of the *vsp*s. Clades and subgroups within clades were identified for various lengths of upstream sequence, all anchored with the 3' conserved region (data not shown). The first minor clade analyzed for recombination involved a group of *vsp *genes with similarity along the 234 bp comprising the 3' terminus. In the phylogenetic tree, these 12 *vsp *sequences actually separated into two related minor groups or branches (all within Clade II of the three major clades of *vsp *genes; see Alignments and phylogenetic tree analysis section and Fig 2). The percent polymorphism for these 12 genes as a group was 74.36% (Table 1). The two subgroups consisted of eight and four *vsp*s genes with 64.52% and 25.21% polymorphisms, respectively (Table 1). Recombination analysis demonstrated significant recombination at Bonferroni corrected values of 0.0311 and 0.0071 respectively, within each of the two subgroups. No significant recombination could be detected between the group of eight and the group of four genes.

**Table 1:** Recombination analysis

DNA polymorphism analysis					Sawyers test for recombination							
GROUP	Number of VSPs	BP	GAPS	POLYMORPHISMS (%)	SINGLE VAR SITES	Pi-Value	Inner frags	Best inner p-val	Best inner BC-val	Outer frags	Best outer p-val	Best outer BC-val
All (1&2)	12	234	0	174 (74.36)	42	0.3403	26	0.0000	0.0007	0	N/A	N/A
clade1	8	234	0	151 (64.52)	85	0.2599	16	0.0000	0.0002	1	0.0027	0.0311
clade2	4	234	0	59 (25.21)	50	0.1467	4	0.0008	0.0073	4	0.0011	0.0071
												
longClade	13	657	0	153 (23.28)	17	0.0865	41	0.0000	0.0000	1	0.0365	0.3755
												
All (3 & 4)	7	546	0	143 (26.19)	14	0.1347	10	0.0000	0.0002	1	0.0013	0.0114
clade3	3	546	0	95 (17.4)	95	0.1160	2	0.0000	0.0002	2	0.0000	0.0002
clade4	4	546	0	110 (20.14)	72	0.1178	6	0.0048	0.0268	2	0.0195	0.1069

Clade 1 contains vsp genes; 41, 180, 49, 48.1, 204, 31, 43, and 38.

Clade 2 contains vsp genes: 95, 18, 21 and 23

longClade contains vsp genes: 279, 235, 272, 111, 248, 223, 191, 83, 234, 271, 222, 216 and 137

Clade 3 contains vsp genes: 217, 139 and 138

Clade 4 contains vsp genes: 270, 261, 290 and 62

Clade 5 contains vsp genes: 150, 140, 153 and 151

Clade 6 contains vsp genes: 245, 82 and 210.

The longest sequence that we could find extending upstream from the 3' terminus, with a sufficient number of *vsp *genes containing related sequence was 657 bp, found in 13 genes. These 13 *vsp *genes demonstrated 23.28% polymorphism and showed significant recombination between them (p = 0.0365) in this region.

We then asked if by shortening the upstream sequence down from 657 bp, whether we could find minor clades containing at least two subgroups of *vsp*s that could then be tested in a manner similar to what was attempted for the first 234 base pair region studied (in other words, recombination across sub-groups). Shortening the sequence down to 546 bases allowed us to find seven *vsp *genes that separated into two subgroups of three and four members each. Here, a Bonferroni-corrected (BC) P-value for recombination was found within the clade of seven sequences (0.0114), demonstrating evidence for recombination across the two minor groups. Also, evidence of recombination was found within the individuals of the three member clade (0.0002 BC-corrected) and within the four member subgroup (0.0195). The Dualbrothers recombination algorithm confirmed the recombination data between these seven members and allowed discovery of several high mutation frequency regions at ~25 bp and 250 bp (Additional file 6A). These high frequency mutation regions could be bracketing DNA fragments and assisting in their horizontal movement, while keeping the intervening sequence intact. A 552 base pair fragment was also discovered for seven other *vsp *genes, and in this analysis, BC values of .0001 for the seven members again demonstrated recombination across two minor subgroups. In addition, a 0.0000 value within the four member group demonstrated recombination within this four member subgroup. Within the three member subgroup, no recombination could be detected, probably due to the low amount of sequence polymorphism observed across these three members (7.24%). Dualbrothers analysis confirmed evidence of recombination across the 552 bases of these seven genes (Additional file 6B). In summary, evidence exists that recombination has occurred within conserved but divergent regions of the *vsp *genes. Evidence also exists that discreet high-mutation-frequency regions bracket low-frequency regions, implying protection or movement of functionally important regions during recombination.

### Alignments and phylogenetic tree analysis of 218 complete VSP proteins and *vsp *DNA sequences

The 218 full length *Giardia *WB VSP proteins were aligned and an unrooted tree produced (Fig 2). The majority of the 218 VSP protein sequences fall into three, well-defined clades labeled I, II and III (Fig 2). Three VSP proteins, whose branches are colored black (*vsp*s3, 13, and 122) lie between clades II and III and contain sequences common to both of those clades. This tree appears to suggest an expansion of three functional groupings of VSP proteins within the WB genome. Of the 30 *vsp *genes containing tandem repeats, 27 fell within clades II and III, while the remaining three consisted of the three *vsp *genes that were between clades II and III. We also performed a protein alignment using two copies of the tandem repeats, taken from each of the 30 TR-containing genes and discovered that they clustered into several distinct clades (Additional file 2). Since a previous report described a *vsp *gene (*vsp*G3M/*vsp*A6-S1; *vsp*175) that was highly similar along the entire gene with *vsp*A6 (*vsp*67), but contained only one copy of the 195 bp repeat [19], we theorized that degenerated sequence forms of the conserved tandem repeats may exist within non-repeat-containing *vsp *genes. We performed a BLAST analysis using single copies of each of the 30 TR to query the non-repeat-containing *vsp *genes. Nine of the 30 demonstrated >80% nucleotide identity over a stretch of at least 80 bp to 10 of the non-repeat-containing genes. This result suggests that additional divergent homologies exist, that other *vsp *genes may have contained similar repetitive motifs that diverged over time, or that recombination between repetitive motifs and non-repeat-containing *vsp *genes might be occurring.

In order to compare protein to DNA phylogenetic relatedness, and to determine if the DNA sequences differed substantially from the protein sequences, the DNA sequences encoding these 218 *vsp *genes were aligned and a Neighbor Joining tree with boot strap values at significant nodes was produced (Additional file 5). A color coding scheme illustrating the three dominant clades produced from the protein alignment along with those associated numerical clade designations is shown in Additional file 5. Much of the conservation and clade designation seen in the protein-based tree is apparent in the DNA sequence-based tree. Further characterization and finishing of the partial *vsp *sequences will determine if these predominant grouping trends hold true for all *vsp *genes and their protein encoding sequences within the WB genome.

### Protein motifs may imply potential VSP function

The protein sequences of all complete *vsp *genes were analyzed for the presence of motifs that might provide clues to their function. A zinc finger motif has been described as being encoded by a subset of the *vsp *genes [38], and zinc binding has been documented for VSPs, although in substoichiometric amounts [39]. Therefore, we were especially interested to see how commonly the zinc finger motif was found across the entire repertoire of 218 complete WB VSPs. Using Pfam 22.0 to search the 218 complete VSPs for protein motifs, we found 36 VSPs that had weak hits (E value between 1 and 1e-5) to the C3HC4 type zinc finger (RING finger), which is a cysteine-rich domain of 40-60 residues, has the consensus sequence: C-X2-C-X(9-39)-C-X(1-3)-H-X(2-3)-C-X2-C-X(4-48)-C-X2-C where × is any amino acid and coordinates two zinc ions. Since we found only weak hits to a Zinc RING finger motif, we hypothesized that the VSPs contain a novel *Giardia*-specific Zinc finger. To elucidate this further we searched for the previously described motifs [39], namely, CxxCHxxCxxC and CxxCxxxCxxC. Table 2 shows data for the number of these two motifs found within the 218 complete WB VSPs and the alternative amino acids found in the 5^th ^position of the CxxCxxxCxxC motif. One or both of these motifs was present in 151 vsps with 73 copies of the CxxCHxxCxxC motif in 67 VSPs and 255 copies of the CxxCxxxCxxC motif present in 149 VSPs. Only two vsps had the CxxCHxxCxxC, but not the CxxCxxxCxxC motif. More experiments are needed to determine if the CxxCHxxCxxC and/or CxxCxxxCxxC motif is required for zinc binding or if there is a difference in their zinc binding properties. Interestingly, the CxxCHxxCxxC motif does not seem to overlap with the weak RING finger domain identified by Pfam, providing further evidence of a putative novel zinc or other metal, or other substrate-binding domain within those VSPs.

**Table 2:** Zinc finger motifs

Fifth AA	#	Charge
H	73	basic

N	60	polar

D	54	acidic

G	50	polar

A	33	nonpolar

P	18	nonpolar

S	17	polar

T	15	polar

V	5	nonpolar

Q	2	polar

E	1	acidic

Total	328	

Of the 218 complete VSPs, 182 (83%) have a GGCY motif. The average number of GGCYs in these 182 genes is 1.4 per VSP, with a maximum of five occurring in one *vsp*. Most GGCY motifs are in the carboxyl portion of VSPs but some exist closer to the N terminus. The function of the GGCY motif is not known, some data suggest that it may be immunogenic. For example, a 12 amino acid epitope containing this motif induced late appearing antibodies in neonatal mouse infections [40], and antibodies to a larger fragment containing the GGCY motif caused trophozoite detachment and aggregation [41].

In addition to the weak RING finger domain, three VSPs had very good (E value <1e-20) hits to the BmKX domain, a short bioactive peptide present in the venom of scorpions and thought to act as a potassium channel blocker [42]. Five additional VSPs showed moderate (E value between 1e-5 and 1e-20) hits to the Lamin_EGF domain (2 VSPs) and the EGF_CA domain (3 VSPs). Many other VSPs had weak hits to these three domains and several additional domains (TIL and Furin-like) that are also cysteine rich. Whether these similarities are of biological significance remains to be determined, but it is interesting to speculate that some of these data implies diversity of *vsp *function.

The VSPs share a high degree of similarity of approximately 38 amino acids encoded by the 3' terminus of the genes. Hidden Markov Modeling (HMM) to identify membrane-spanning regions suggested that the C-terminal CRGKA motif is inside the membrane, while the 23 amino acid upstream and adjacent to the CRGKA span the membrane, and the remainder of the VSP is extracellular (data not shown). This result is consistent with prior hydrophilicity analyses [17]. As previously discussed above, the CRGKA encoding C-terminal tail was used to identify all known *vsp *members within the current WB genome assembly and is found in all reported *vsp *genes, suggesting an important function in VSP biology. Many VSPs are palmitoylated [43,44], and in vivo, palmitic acid binds specifically to the cysteine of the CRGKA tail controlling segregation of the VSPs to the detergent-resistant domains within the plasma membrane [45]. Arginine deiminase binds specifically *in vivo *to the arginine of the CRGKA and catalyzes its citrullination [46]. These biochemical studies in addition to the highly conserved nature of the CRGKA motif suggest an important biological role. Thus, it is of interest that eight *vsp*s have amino termini that differ from the CRGKA at one or two positions; CHKKA, CRGKL, CRSKA, GRRKA (2 examples), FCGKA, YRGKA, and WRGKA (Additional file 1), and six of these eight lack either the cysteine or the arginine or both. Experimental studies will be required to determine whether these alternate N-termini are compatible with observed or hypothesized VSP function.

### Genomic organization and context of the *vsp *genes in the WB genome

**Figure 3:**
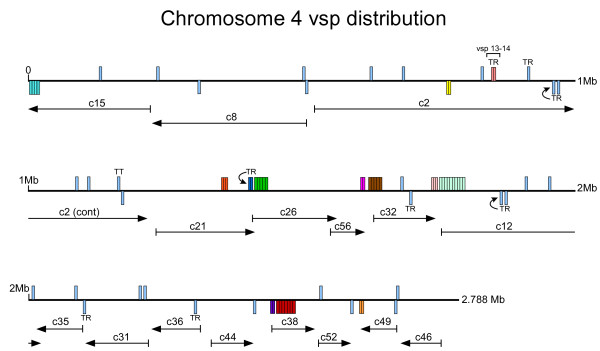
**Distribution of *vsp *genes along chromosome 4**. The contigs were initially assigned to individual chromosomes by mapping end-sequenced BACs to PFG separations of chromosomes and subsequently refined by optical mapping. Chromosome 4 is the most densely populated with *vsp *genes (29/Mb). Each *vsp *gene is noted by a vertical line, which is above the horizontal chromosome line for those with a forward orientation in relationship to the chromosome direction and below for those oriented in a reverse direction. The contigs from Assembly 14 [30] are shown below with arrows indicating the orientation of the contig. *Vsp*s with tandem repeats are noted with "TR" and those present in linear arrays are shown as filled in boxes with each section representing one *vsp *gene. TT indicates tail to tail arrangement.

**Figure 4:**
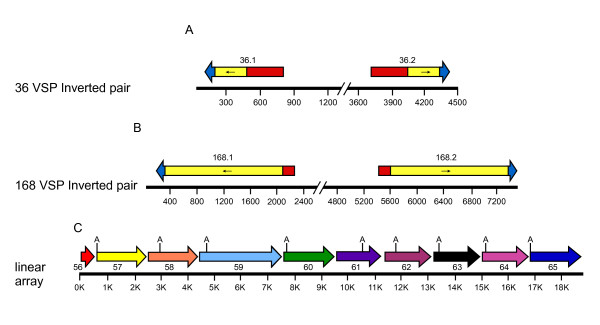
**Patterns of genomic organization of *vsp *genes**. Genes present as inverted pairs are shown in A and B. These are in tail to tail orientation, and overall there were 10 paired identical tail to tail dual gene sets and 4 paired identical head to head dual gene sets. A linear array of *vsp*s 57 to *vsp*65 is shown in C. Gene 59 is a clade II representative while the other *vsp *genes (57, 58, and 60-65 are all Clade I representatives.

The *vsp *genes were distributed among all five chromosomes (Table 3). Of the 196 *vsp *genes that could be assigned to a specific chromosome, the density ranged from 8 *vsp *genes per Mb on chromosome 1 to 29 *vsp *genes per Mb on chromosome 4 (based on the total size determined by optical mapping). The distribution of vsp genes along chromosome 4 is shown in Fig 3. Many of the *vsp *genes existed independently on the chromosome with no other *vsp *genes in the near proximity. However, the majority of *vsp *genes were not randomly distributed, but were clustered into head to head or tail to tail arrangements, or as linear arrays (LAs) of *vsp *genes (see Fig 4 for examples).

**Table 3:** *Vsp *chromosomal distribution

Chromosome (size Mb)	*vsp*s	#/Mb
5 (4.43)	65	15

4 (2.79)	81	29

3 (1.94)	18	9

2 (1.50)	20	13

1 (1.46)	12	8

### Genes present in inverted or tandem pairs (H-H, T-T, H-T)

*Vsp *gene pairs in *Giardia *can appear as an inverted pair of genes with or without identical reading frames (Table 4). Fourteen of the 19 inverted pairs consisted of two identical ORFs. Conversely, there were no examples of identical genes that were not in this inverted pair arrangement. The one example reported in the literature, (*vsp1267*), consisted of two identical ORFs in tail to tail orientation, separated by approximately 3 kb [17]. In the current study, we discovered 13 additional pairs of identical genes present in head to head (4) or tail to tail (9) configurations with 2190 to 3847 bp of intervening DNA between the paired ORFs (Table 5). Outside the coding regions of these identical pairs, the 5' and 3' flanking sequences of the two members of a pair were nearly identical up to a point of abrupt divergence which ranged from 139 bp to 729 bp away from the coding regions (Table 5).

**Table 4:** *Vsp *gene patterns

Pattern	All *vsp*s	Complete sequences only
Inverted identical pairs	14 pairs	

Head to head	4	4

Tail to tail	10	10 (17 genes, but 3 of the paired members were incomplete)

Other head to head	10 (5 pairs)	10 (5 pairs)

Other tail to tail	4 (2 pairs)	3 (2 pairs)

Linear array	164	99

Overlapping linear array	5	5

		

Total *vsp *genes	303	228

**Table 5:** Divergence points of identical pairs of genes

Gene #	5' Divergence (nt above start codon)	3' Divergence (nt beyond stop codon)	Distance between ORFs
Head to head			

26	169	-	2190

36	729	203	3102

162	561	-	3847

168	158	141	3302

Tail to tail			

1	-	559	2814

7	-	318 (572)	2687

20	-	541	2759

47	-	354	2721

48	-	515	3222

53	-	486	2670

87	-	514	2393

107	-	529	2638

126	-	530	2748

The occurrence of identical pairs of genes was especially problematic during genome assembly in that this redundancy prematurely terminated the contigs. In fact, *vsp*1267 (*vsp*98.1) was not identified as a pair by the genome assembly, but was found as a single gene at the end of a contig. Only two of the 14 pairs with identical members were greater than 10 kb away from the end of any given contig. Whether other inverted pairs were missed by the assembly is not known but quite possible.

An additional five pairs of genes where the two paired members contained different ORFs were discovered in head to head (4) or tail to tail (1) orientation and were separated by approximately 3 kb of intervening DNA in all cases. For four of these five pairs, the two members of the pair were from different clades, implying translocation from other perhaps singular genomic locations into this tandem arrangement. We believe that the 14 identical pairs have arisen purely by gene duplication and transposition events. It is possible that these gene duplication events are relatively recent in the evolutionary history of the *vsp *gene family and that over time, genetic diversity through recombination between identical or distant *vsp *genes may produce new *vsp *genes. The paired, identical *vsp*s and non identical paired *vsp*s remain an interesting, but cryptic observation of the *vsp *gene family.

### *vsp *genes present as linear arrays (LA)

Over half the *vsp *genes found in the genome lie within the genomic context of *vsp *linear arrays that consist of two or more adjacent *vsp *genes (Table 4). The intervening regions between the genes in these linear arrays averaged 60 bp (ranging from overlapping encoded regions to 241 bp of intergenic sequence). Apart from these linear arrays, there were only two other head to tail pairs of *vsp *genes that were less than 5 kb apart, but containing greater than one kb of intergenic sequence between the two genes. For these two non-linear array pairs, the two members of a pair had no significant sequence similarity. Therefore, this paired association may be random.

Most of the linear arrays were in very short contigs, such that they frequently included only two *vsp *genes, and often terminated one or both ends of the contig on which they were located. In fact, 97 of the 102 *vsp *genes in contigs <10 kb in length were in linear arrays. In terms of the contigs themselves, *vsp *linear arrays accounted for the majority of coding capacity of 56 of the contigs < 10 kb in length, even after some were joined by directed, manual sequencing efforts. In the larger contigs, there are several examples of linear arrays at the ends of adjacent contigs (see Fig 3), suggesting that these adjacent linear arrays may indeed represent even longer arrays than what is observed here. The possibility of longer arrays also is supported by the observation that the longest array discovered to date (10 *vsp *genes) is on the end of contig 12 that is adjacent to the end of contig 32 (optical mapping data not shown), which has two *vsp *genes in a linear array (Fig 3). Therefore, while the *Giardia *linear arrays described here are impressive in their quantity, length and gene numbers, much longer arrays may exist in the genome.

As noted above, the first *vsp *of a linear array often showed the typical features of *vsp *genes that have been described in the literature, including an extended noncoding region upstream and a putative initiator element (Inr). Fig 3 shows the spacing within the linear array and the variable placing of the first ATG after the nearest upstream stop codon. In fact, *vsp*14 provides an example of a sequence that contains a typically conserved 3' region, but which lacks an initiation codon. *Vsp*13 is the first member of a linear array on chromosome 4. The coding region for *Vsp *13 is followed by a stop codon, 38 bp, another stop codon, and 224 bp of coding region for *vsp *14, which also contains the conserved encoded CRGKA, C-terminal domain. This short, stop-containing intergenic region suggests that *vsp *13 may not be expressed. The frequency of these linear array formations along with the lack of an Inr sequence in these downstream members and the occurrence of short, intergenic or overlapping encoding regions between these downstream *vsp *genes leads us to hypothesize that these downstream members may not be actively expressed, either individually or in an operon-like fashion. A potential hypothesis for their occurrence or genesis is that they may serve as cassettes for recombination with the first *vsp *gene in the linear array (that contains functional upstream expression sequence) or distant, expressible *vsp *genes in the genome. In both scenarios, the generation of new antigenic or functional *vsp *genes would be the potential outcome of such a scenario. Pursuant to this hypothesis, we discovered that in certain linear arrays, members within a linear array were sometimes more closely related to each other phylogenetically than to *vsp *genes elsewhere in the genome, or in other linear arrays (data not shown). We also discovered that for some linear arrays, members within the array were far more phylogenetically distant to each other than they were to genomically distant *vsp *genes (data not shown) suggesting either active recombination to generate significant sequence diversity or translocation of entire *vsp *coding regions into linear array formations. Except for 13 of the 26 linear arrays containing at least two complete genes, all members of a linear array are from the same phylogenetic clade (Data not shown). The longest array (Fig 3) had nine complete sequences, and eight of these *vsp *genes were from Clade I while one *vsp *gene was from Clade II (Fig 4C). Among the eight Clade I *vsp*s in this linear, there were two pairs (*vsp*60/65 and *vsp *61/64) in which the two members of each pair were the closest relatives to each other on the phylogenetic tree, implying previous gene duplication, but little to no distant translocation events. For example, *vsp*s61 and 64 demonstrated 94% nucleotide sequence identity, including long regions of 100% identity. These closely related pairs suggest that gene duplication and divergence have played a role in expansion of the linear arrays. In addition, sequence drift and recombination-driven sequence divergence may have also played roles in the formation of these arrays. We speculate that these linear arrays contain genes that are nonfunctional, but either provide a reservoir for expanding the *vsp *gene repertoire, or alternatively, represent the remains of discarded *vsp *genes.

### Rarity of telomeric *vsp *genes

For other eukaryotic, non-*Giardia *microbial pathogens, variant surface antigen genes are present and are frequently located in sub-telomeric locations. For example, the VSG genes of African trypanosomes have been the most intensively studied variable surface antigen genes and they can be subtelomeric or chromosome-internal in their location. Trypanosome chromosome-internal VSG genes are expressed only when they are duplicated into subtelomeric expression locations [47,48]. An additional example of variable surface antigen genes can be found in *Plasmodium falciparum *parasites which express one of a family of *var *genes that encode proteins that are involved in adherence to host cells. Variation in expression from one *var *gene to another can change the binding characteristics of the organism. The *P. falciparum *genome has been completely sequenced and assembled, and the 61 *var *genes have all been assigned to their chromosomal locations. While the *var *genes can be chromosome-internal, nearly two thirds of them are subtelomeric [49]. Activation of subtelomeric or chromosome-internal *var *genes occurs by epigenetic mechanisms in the absence of DNA recombination [49,50].

In contrast, *Giardia*'s *vsp *family appears to be very different from the trypanosome VSG or plasmodium *var *gene families. First of all, only three *vsp *genes have been found in subtelomeric locations, most likely, all at one end of chromosome 5 [51]. In addition, there is no evidence for duplicative transposition of *vsp *genes to subtelomeric or other locations as a requirement for *vsp *gene expression in *Giardia*. As an example, for two different *vsp *genes (*vspA6 *and *vspC5*), the chromosomal locations have been determined in organisms expressing these genes and in antigenic variants that have lost expression of these genes. In both cases, the chromosomal location did not change with antigenic variation [10,11], and in both cases, the *vsps *were chromosome-internal. Thus, *Giardia *differs from these other protists in that a subtelomeric location for the *vsps *is relatively uncommon, and is clearly not required for expression.

## Conclusions

This description of the vsp genes and their encoded proteins from the WB isolate gives a picture of a repertoire that has been expanded by duplication and recombination to the extent that expression of different VSPs may facilitate differences in pathogenesis or ecological niches. This description will provide important tools for better understanding of the biology of these enigmatic proteins and the trophozoite surfaces on which they are expressed.

## Methods

### *Vsp *gene identification

Identification of *vsp *genes in the WB genome was performed by a combination of three approaches, (1) Called ORFs that were identified by the automated BLAST search as variant-specific surface proteins or trophozoite surface antigens, (2) called ORFs with at least two CXXC motifs in the encoded sequence, and (3) BLAST searches of the genome assembly with the conserved 3' 110 bp. Overall, the third approach was the most efficient at identifying all *vsp*s except those ORF-coding regions where the sequence was incomplete and lacking the 3' conserved regions. Another set of cysteine-rich genes has been identified in *Giardia *(HCMP), which also have CXXC motifs, occasional CXC motifs that are not found in the *vsp *genes, but which collectively lack the 3' conserved region [36]. Therefore, we have compared the lists of identified *vsp*s with other cysteine-rich genes to eliminate the possibility of dual category assignment of genes. Ultimately, the presence of the CRGKA motif at the C-terminus was the defining feature for *vsp*-designated genes; for all *vsp *designated genes three of the five encoded amino acids of the CRGKA motif must be present in the 3' coding end of the gene.

### Genomic mapping and chromosomal localization using the BACs, extensive chromosomal hybridizations, and optical mapping

DNA sequences from individual clones were assembled into contigs by ARACHNE after editing with CONSED at described [30]. The assembled contigs and supercontigs were compared directly to the BAC scaffolding. Many of the BACs had been labeled and hybridized to PFGE blots of *Giardia *chromosomes, allowing placement of the supercontigs onto specific chromosomes. The chromosome location of contigs has subsequently been verified and expanded upon by data generated from the use of optical mapping technologies. For optical mapping, whole chromosomes from Giardia lamblia WBC6 were isolated from trophozoite cell pellets using pulsed field gel electrophoresis. Agarose plugs containing the chromosomes were sent to OpGen (Gaithersburg, MD) for mapping. Optical mapping was done with MluI restriction enzyme and the observed fragments compared to the MluI sites predicted in the assembled contigs using MapSolver v. 2.1.1. Sixty-two contigs representing 10.4 MB uniquely mapped to one of the five chromosomes.

### Directed sequencing

Since many of the contigs were prematurely terminated at or near *vsp *sequences, we also subjected some of the clones that aligned to these problem ends of contig regions to directed sequencing. These extended clone sequences were then used to join or extend contigs when possible and to extend or complete *vsp *gene sequences. In addition to the libraries used for the genome project, an additional library was constructed from WB DNA that was partially digested with SauIIIA1, cloned into pUC18, and hybridized to the *vsp *3' conserved region. Approximately 670 clones were subjected to vector-junction sequencing. Following analysis, additional directed sequencing was performed for 308 clones that had the potential of adding to the existing *vsp *sequence data.

### Search for motifs

The VSP amino acid sequences were analyzed using Pfam 23.0 [52] to search for Zinc finger motifs. An e-value cutoff of 10 was used to capture VSPs with weak hits to Zinc fingers. Very weak Zinc finger domains were identified in 181 VSPs.

### Alignments of *vsp *genes and recombination testing

Full length VSP proteins and the N-terminal 38 amino acids for all VSPs were aligned using MUSCLE 1.1. Full length *vsp *genes, the 3' 117 nucleotides for all *vsp*s, and two of the tandem repeat encoding sequences of the tandem repeat-containing genes were aligned using CLC Genomic Workbench 3.2 (Katrinebjerg, Denmark). Alignment trees were constructed using the Neighbor Joining method with 1000 bootstraps. The alignments were transferred into MacClade program (Maddison D.R. and W.P. Maddison. 2003; MacClade 4 analysis of phylogeny and character evolution, version 4.06 Sinauer Associates, Sunderland Mass.) and manually corrected. DNA polymorphism analysis and recombination analysis were performed with the DNasp package of algorithms http://www.ub.es/dnasp. Sawyer's test was performed with GENECONV http://www.math.wustl.edu/~sawyer/mbprogs/ as previously described [53]. Additional recombination analysis was performed using the Dualbrothers algorithm [54].

## Authors' contributions

RA coordinated the project and writing and did a portion of the directed sequencing. AN compiled and analyzed vsp sequences. VS compiled and analyzed vsp sequences. CM performed bioinformatics and sequence searching and comparison analysis. GF performed vsp Recombination analysis. HM provided the optical mapping data. TN directed sequencing of additional vsp-containing clones and assisted with the analysis and writing. SP coordinated directed sequencing efforts and analyses including recombination, motif and phylogenetic analyses. RP worked on compiling and analyzing vsp sequences. All authors read and approved the final manuscript.

## Supplementary Material

Additional file 1 **List of all *vsp *genes with detailed information as follows: A. VSP number, B. Clade number (of the three major clades)**. A blank indicates either an incomplete sequence or that this is one of the three genes without a clade assignment, C. Name used in prior reports in the literature, D. Prior Genbank number, when available, E. gene # from the WB genome (the number that is given should be preceded by the prefix GL50803_), F. Features including TT (tail to tail), HH (head to head), IP (inverted pair of identical ORFs; the "a" or "b" refers to the first or second member of the pair, TR (tandem repeats; the following number indicates the number of nucleotides per repeat in the reported sequence, LA (linear array; it is followed by O for overlapping genes, G. An "x" is used to note the members of linear arrays that were first in the array, H. Number of nt/tandem repeat, I. Rep # refers to the number of tandem repeats, J. Chromosomal location, K. Compl refers to whether the sequence contains the complete open reading frame; c indicates complete, 3 indicates missing 3' sequence and 5 indicates missing 5' sequence, L. Inr indicates whether or not a gene contains the putative Inr and whether it is a TAATGTT or CAATGTT, M. Alternative C-terminal 5 amino acid motif, in place of CRGKA, N. number of amino acids in the translated sequence, O. The molecular mass of the putative protein in kDA, P. The number of cysteines in the encoded sequence, Q. % cysteines in the encoded protein, and R. Number of CXXC motifs in the encoded protein. Click here for file

Additional file 2 **Rooted phylogenetic tree of the DNA sequences of the 30 tandem repeat regions**. Two of the tandem repeat DNA sequence domains from all of the known and complete tandem repeat-containing genes were aligned and a Neighbor Joining method tree was constructed with 1000 bootstraps. Names of the respective tandem repeat-containing genes are shown where TR stands for Tandem Repeat. This tree suggests that some commonality and difference of the tandem repeats exist relative to each other, implying sequence divergence or possible recombinatorial actions driving the variety of TRs. Click here for file

Additional file 3 **Nucleotide alignment of the 3' regions**. The 3'-terminal 117 nucleotides (including the stop codon) for all 218 complete VSPs were aligned and the alignment manually corrected. Colored shading indicates conservation of nucleotides at particular positions across all *vsp*s. At the bottom of the figure is the consensus sequence from this alignment where vertical box height correlates with frequency of occurrence of that nucleotide. Click here for file

Additional file 4 **Amino acid alignment of the 3' regions**. The C-terminal 38 amino acids for all 218 complete VSPs were aligned and the alignment manually corrected. Colored shading indicates conservation of residues at particular positions across all *vsp*s. Dashes indicate absence of a residue. At the bottom of the figure is the consensus sequence from this alignment where vertical box height correlates with frequency of occurrence of that residue. Click here for file

Additional file 5 **Rooted phylogenetic tree of the DNA sequences encoding all 218 full length *vsp *sequences**. Full length *vsp *genes were aligned, manually corrected and a rooted tree created using the Neighbor Joining method with 1000 bootstraps. Colored clade designation along with numerical I, II, and III designations are based upon the unrooted tree shown in Fig 2. The three *vsp*s (*vsp*s 3, 13, and 122) lying between clade II and III are shown in black. The bar at the bottom of the figure signifies branch length related to number of substitutions per base position analyzed. Click here for file

Additional file 6 **DualBrothers recombination alignment of sub clades**. A) Dualbrothers analysis of clade 3 and 4 as defined in the Recombination table. B) Dualbrothers analysis of clade 5 and 6 as defined in the Recombination table. For each panel the x-axis represents the positions in the sequence alignment, the y-axis shows the Bayesian posterior probability of the inferred tree topologies (Topologies) with each colored line indicating a distinct topology (4 shown for A and 3 shown for B), the green line representing the sum of remaining tree topologies. Transition:transversion and expected divergence rates are shown along with an identity bar graph and base positional differences relative to a consensus (colored bars in horizontal grey bars). Click here for file
