# Supplementary figures and images for: The Giardia lamblia vsp gene repertoire: characteristics, genomic organization, and evolution
Source: BMC Genomics. 2010 Jul 9;11:424. doi: 10.1186/1471-2164-11-424 (PMC2996952; doi:10.1186/1471-2164-11-424)

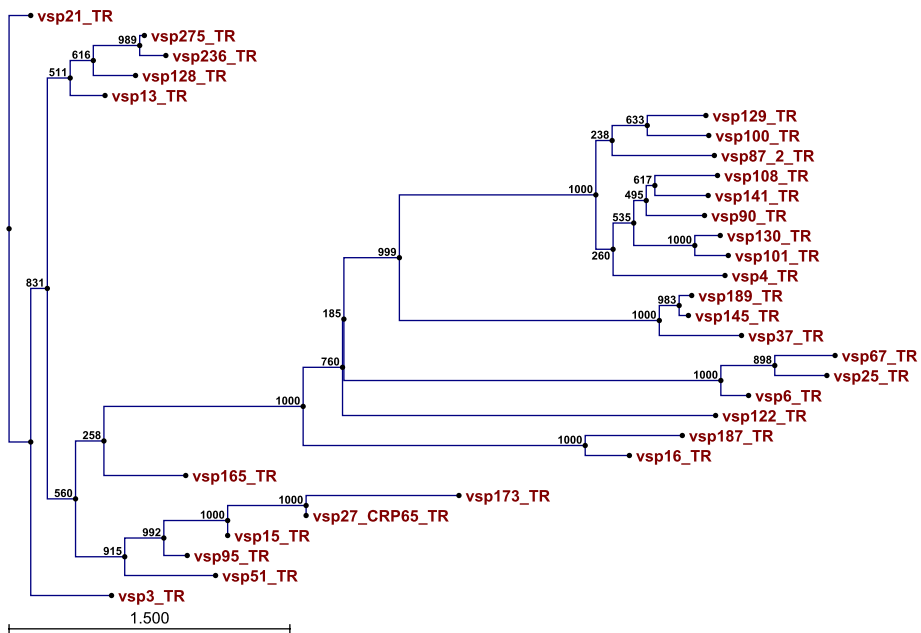

Supplement: Additional file 2 — Rooted phylogenetic tree of the DNA sequences of the 30 tandem repeat regions. Two of the tandem repeat DNA sequence domains from all of the known and complete tandem repeat-containing genes were aligned and a Neighbor Joining method tree was constructed with 1000 bootstraps. Names of the respective tandem repeat-containing genes are shown where TR stands for Tandem Repeat. This tree suggests that some commonality and difference of the tandem repeats exist relative to each other, implying sequence divergence or possible recombinatorial actions driving the variety of TRs. [file 1471-2164-11-424-S2.PDF]

|      | 20        | 40    | 60    | 80    | 100   | 120   |     |
|------|-----------|-------|-------|-------|-------|-------|-----|
| 78   | -----     | ----- | ----- | ----- | ----- | ----- | 117 |
| 40   | -----     | ----- | ----- | ----- | ----- | ----- | 117 |
| 66   | -----     | ----- | ----- | ----- | ----- | ----- | 117 |
| 198  | -----     | ----- | ----- | ----- | ----- | ----- | 117 |
| 190  | -----     | ----- | ----- | ----- | ----- | ----- | 117 |
| 201  | -----     | ----- | ----- | ----- | ----- | ----- | 117 |
| 209  | -----     | ----- | ----- | ----- | ----- | ----- | 117 |
| 73   | -----     | ----- | ----- | ----- | ----- | ----- | 117 |
| 131  | -----     | ----- | ----- | ----- | ----- | ----- | 117 |
| 72   | -----     | ----- | ----- | ----- | ----- | ----- | 117 |
| 221  | -----     | ----- | ----- | ----- | ----- | ----- | 117 |
| 39   | GTGTCTCTG | GCAGC | ----- | ----- | ----- | ----- | 117 |
| 157  | -----     | ----- | ----- | ----- | ----- | ----- | 117 |
| 64   | -----     | ----- | ----- | ----- | ----- | ----- | 117 |
| 124  | -----     | ----- | ----- | ----- | ----- | ----- | 117 |
| 54   | -----     | ----- | ----- | ----- | ----- | ----- | 117 |
| 98.1 | -----     | ----- | ----- | ----- | ----- | ----- | 117 |
| 67   | -----     | ----- | ----- | ----- | ----- | ----- | 117 |
| 175  | -----     | ----- | ----- | ----- | ----- | ----- | 117 |
| 90   | -----     | ----- | ----- | ----- | ----- | ----- | 117 |
| 120  | -----     | ----- | ----- | ----- | ----- | ----- | 117 |
| 243  | -----     | ----- | ----- | ----- | ----- | ----- | 117 |
| 173  | -----     | ----- | ----- | ----- | ----- | ----- | 117 |
| 161  | -----     | ----- | ----- | ----- | ----- | ----- | 117 |
| 236  | -----     | ----- | ----- | ----- | ----- | ----- | 117 |
| 275  | -----     | ----- | ----- | ----- | ----- | ----- | 117 |
| 5    | -----     | ----- | ----- | ----- | ----- | ----- | 117 |
| 10   | -----     | ----- | ----- | ----- | ----- | ----- | 117 |
| 142  | -----     | ----- | ----- | ----- | ----- | ----- | 117 |
| 41   | -----     | ----- | ----- | ----- | ----- | ----- | 117 |
| 42   | -----     | ----- | ----- | ----- | ----- | ----- | 117 |
| 25   | -----     | ----- | ----- | ----- | ----- | ----- | 117 |
| 180  | -----     | ----- | ----- | ----- | ----- | ----- | 117 |
| 26.1 | -----     | ----- | ----- | ----- | ----- | ----- | 117 |
| 127  | -----     | ----- | ----- | ----- | ----- | ----- | 117 |
| 114  | -----     | ----- | ----- | ----- | ----- | ----- | 117 |
| 35   | -----     | ----- | ----- | ----- | ----- | ----- | 117 |
| 183  | -----     | ----- | ----- | ----- | ----- | ----- | 117 |
| 89   | -----     | ----- | ----- | ----- | ----- | ----- | 117 |
| 159  | -----     | ----- | ----- | ----- | ----- | ----- | 117 |
| 181  | -----     | ----- | ----- | ----- | ----- | ----- | 117 |
| 43   | -----     | ----- | ----- | ----- | ----- | ----- | 117 |
| 49   | -----     | ----- | ----- | ----- | ----- | ----- | 117 |
| 101  | -----     | ----- | ----- | ----- | ----- | ----- | 117 |
| 118  | -----     | ----- | ----- | ----- | ----- | ----- | 117 |
| 189  | -----     | ----- | ----- | ----- | ----- | ----- | 117 |
| 182  | -----     | ----- | ----- | ----- | ----- | ----- | 117 |
| 185  | -----     | ----- | ----- | ----- | ----- | ----- | 117 |
| 4    | -----     | ----- | ----- | ----- | ----- | ----- | 117 |
| 113  | -----     | ----- | ----- | ----- | ----- | ----- | 117 |
| 1.1  | -----     | ----- | ----- | ----- | ----- | ----- | 117 |
| 156  | -----     | ----- | ----- | ----- | ----- | ----- | 117 |
| 16   | -----     | ----- | ----- | ----- | ----- | ----- | 117 |
| 27   | -----     | ----- | ----- | ----- | ----- | ----- | 117 |
| 33   | -----     | ----- | ----- | ----- | ----- | ----- | 117 |
| 167  | -----     | ----- | ----- | ----- | ----- | ----- | 117 |
| 53.1 | -----     | ----- | ----- | ----- | ----- | ----- | 117 |
| 48.1 | -----     | ----- | ----- | ----- | ----- | ----- | 117 |
| 130  | -----     | ----- | ----- | ----- | ----- | ----- | 117 |
| 7.1  | -----     | ----- | ----- | ----- | ----- | ----- | 117 |
| 160  | -----     | ----- | ----- | ----- | ----- | ----- | 117 |
| 186  | -----     | ----- | ----- | ----- | ----- | ----- | 117 |
| 88   | -----     | ----- | ----- | ----- | ----- | ----- | 117 |
| 8    | -----     | ----- | ----- | ----- | ----- | ----- | 117 |
| 45   | -----     | ----- | ----- | ----- | ----- | ----- | 117 |
| 34   | -----     | ----- | ----- | ----- | ----- | ----- | 117 |

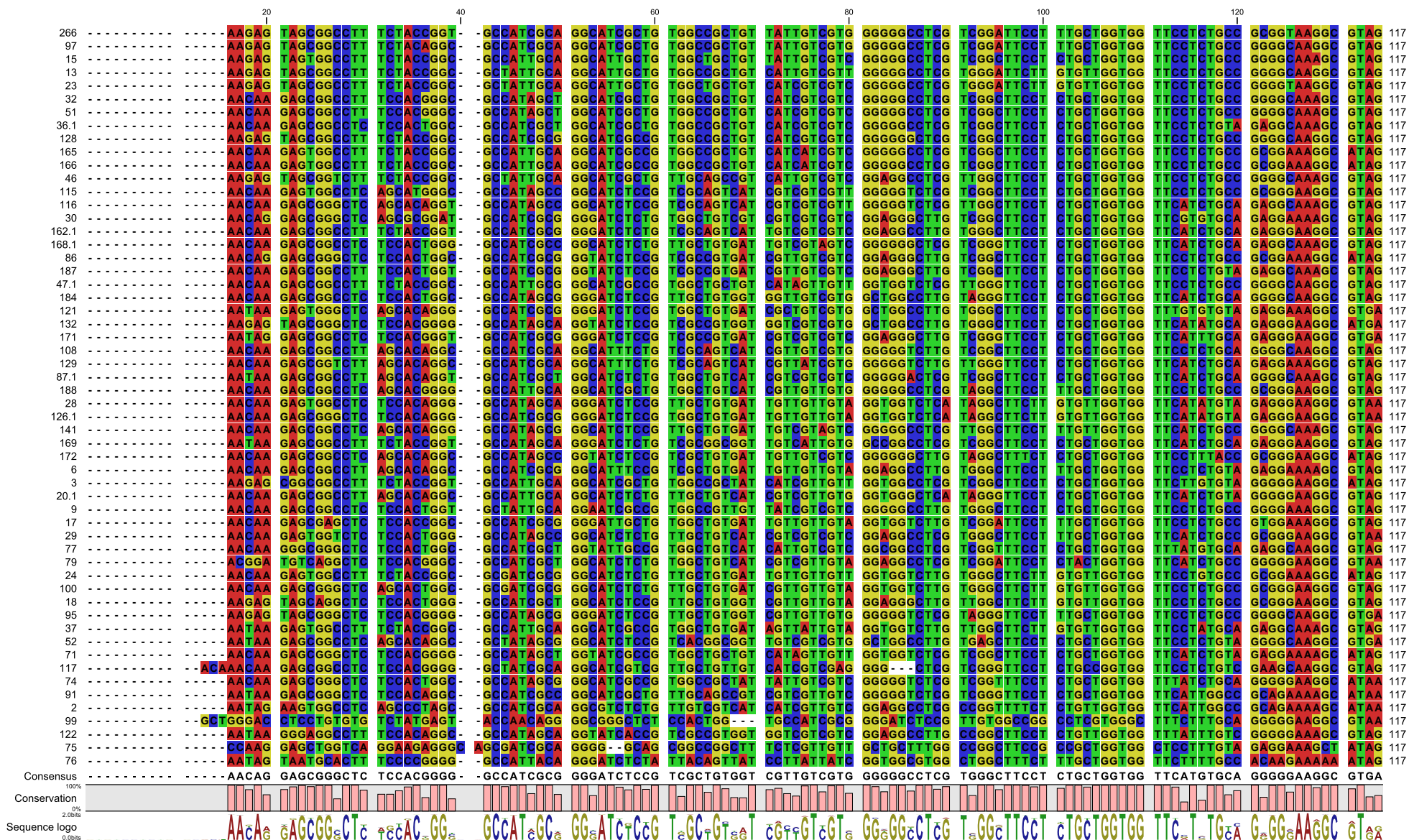

Supplement: Additional file 3 — Nucleotide alignment of the 3' regions. The 3'-terminal 117 nucleotides (including the stop codon) for all 218 complete VSPs were aligned and the alignment manually corrected. Colored shading indicates conservation of nucleotides at particular positions across all vsps. At the bottom of the figure is the consensus sequence from this alignment where vertical box height correlates with frequency of occurrence of that nucleotide. [file 1471-2164-11-424-S3.PDF]

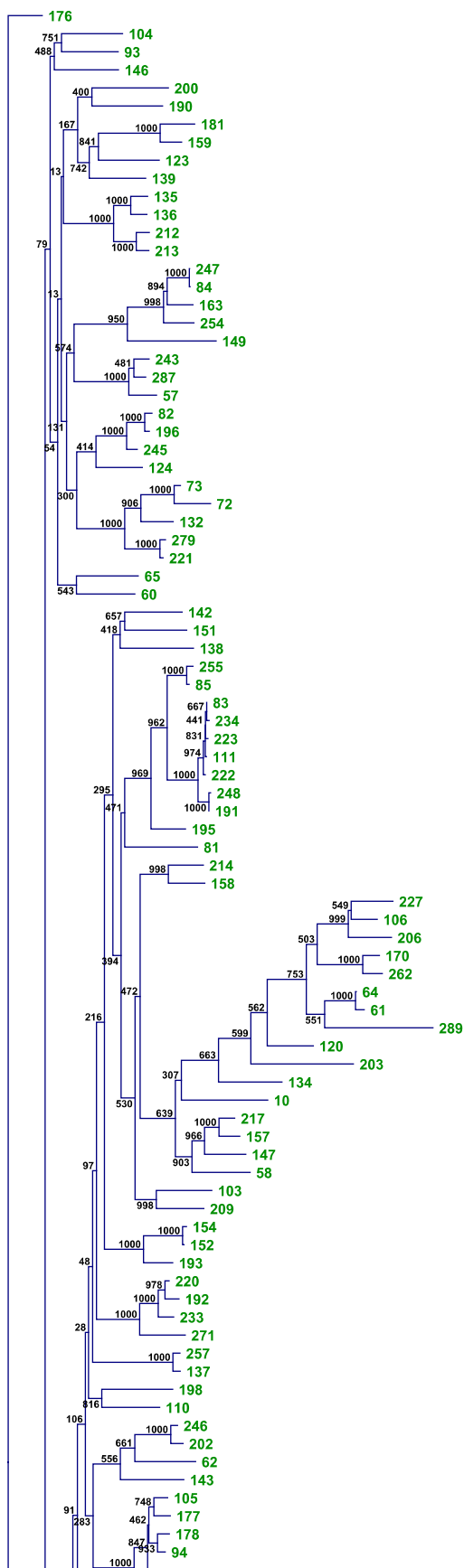

I

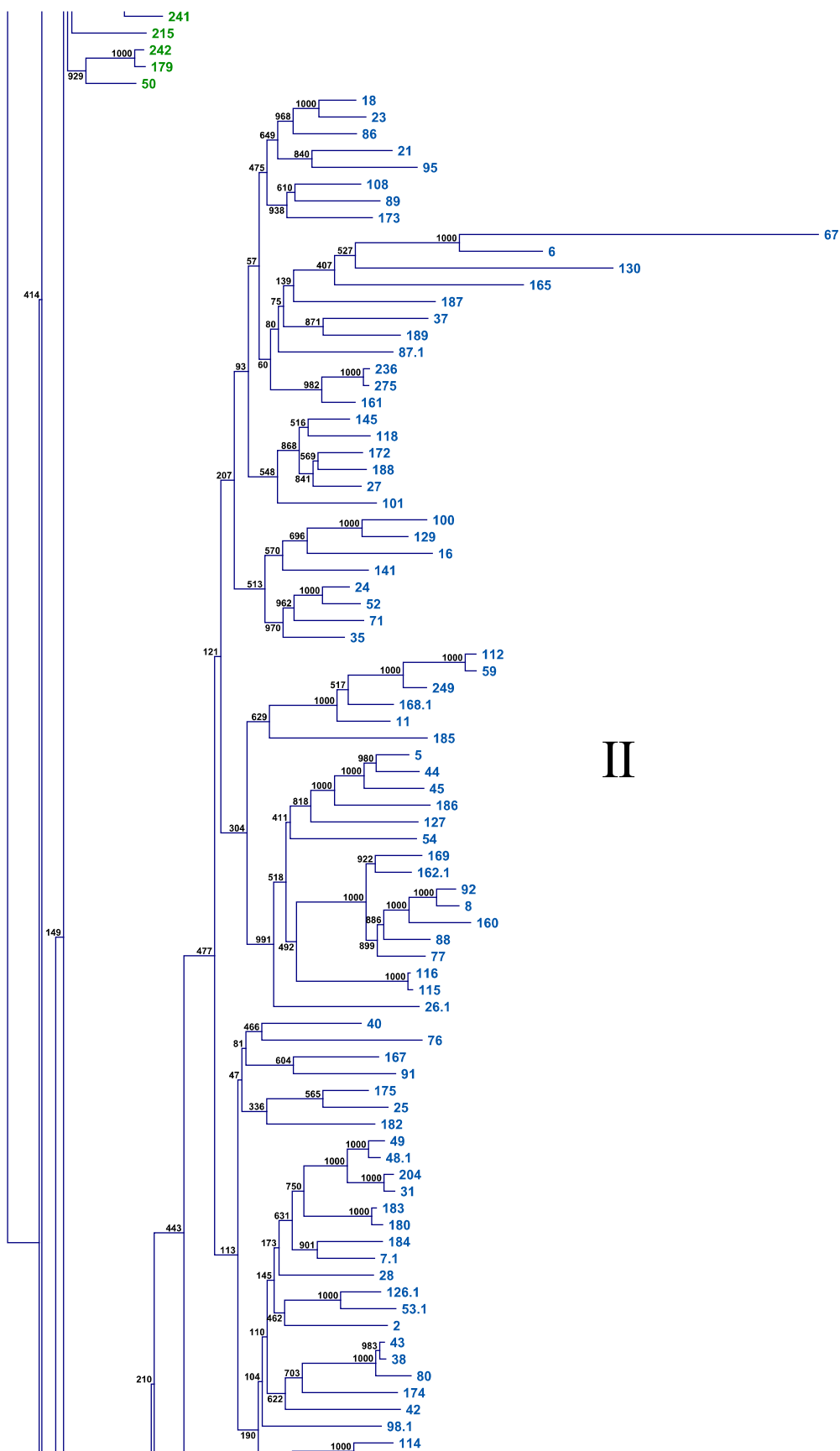

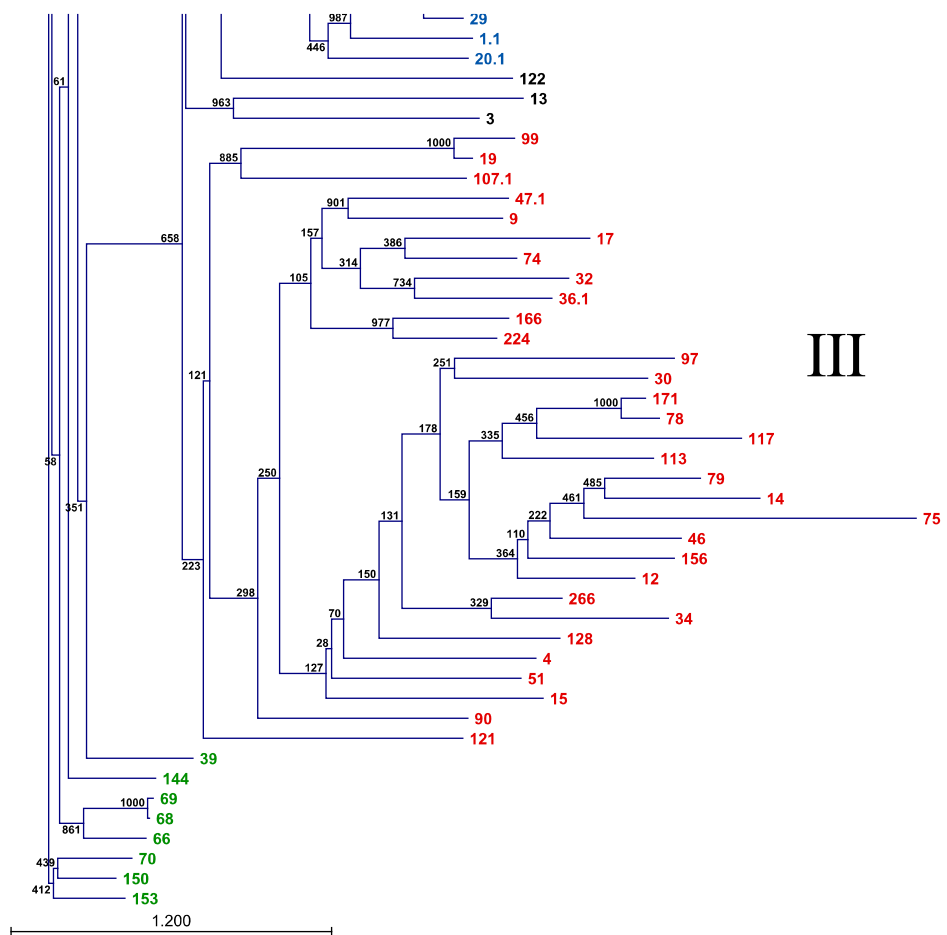

Supplement: Additional file 5 — Rooted phylogenetic tree of the DNA sequences encoding all 218 full length vsp sequences. Full length vsp genes were aligned, manually corrected and a rooted tree created using the Neighbor Joining method with 1000 bootstraps. Colored clade designation along with numerical I, II, and III designations are based upon the unrooted tree shown in Fig 2. The three vsps (vsps 3, 13, and 122) lying between clade II and III are shown in black. The bar at the bottom of the figure signifies branch length related to number of substitutions per base position analyzed. [file 1471-2164-11-424-S5.PDF]

Consensus

Topologies

Kappa (transition:transversion)

Mu (expected divergence)

Identity

1. 270  
2. 290  
3. 62  
4. 261  
5. 138  
6. 217  
7. 139

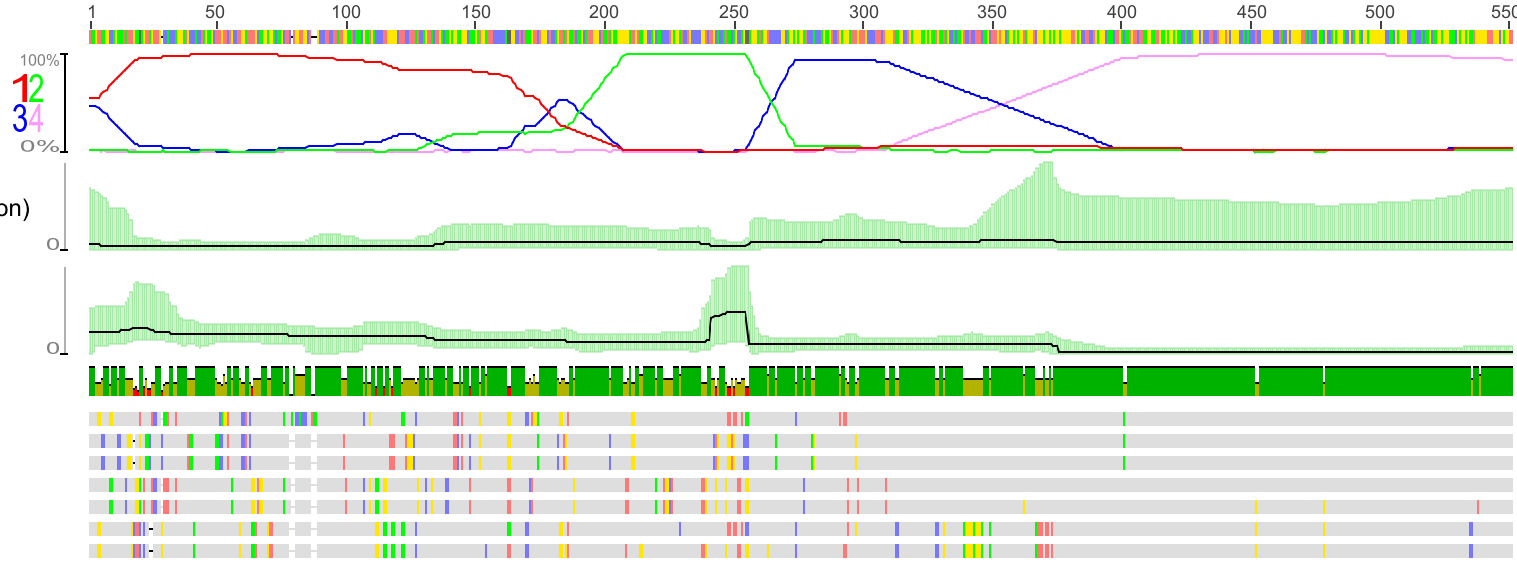

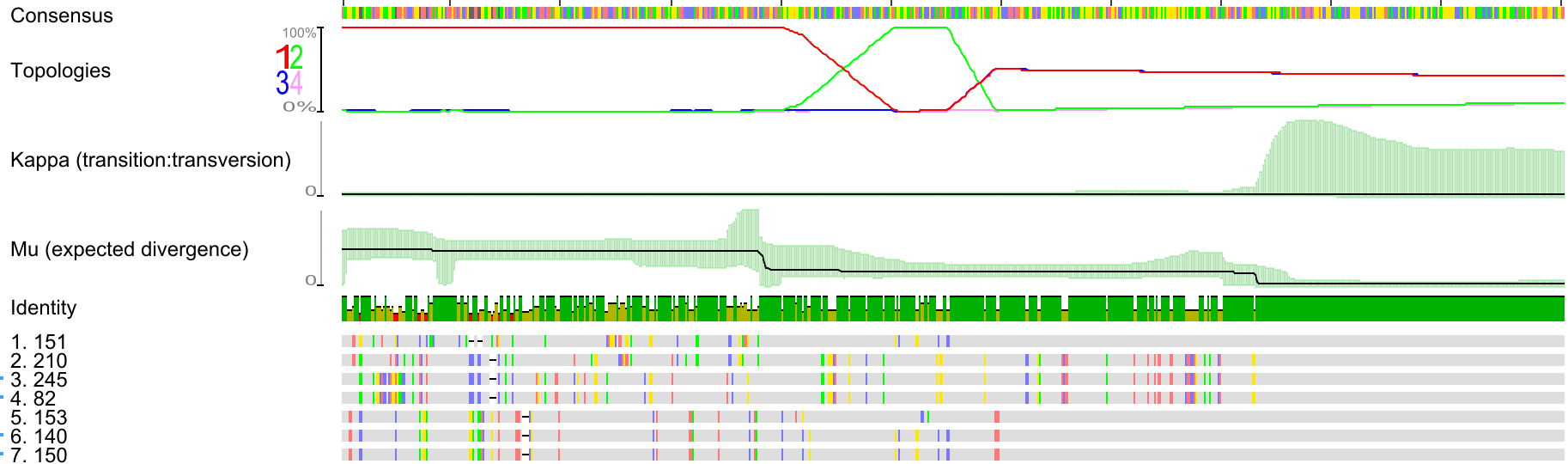

Supplement: Additional file 6 — DualBrothers recombination alignment of sub clades. A) Dualbrothers analysis of clade 3 and 4 as defined in the Recombination table. B) Dualbrothers analysis of clade 5 and 6 as defined in the Recombination table. For each panel the x-axis represents the positions in the sequence alignment, the y-axis shows the Bayesian posterior probability of the inferred tree topologies (Topologies) with each colored line indicating a distinct topology (4 shown for A and 3 shown for B), the green line representing the sum of remaining tree topologies. Transition:transversion and expected divergence rates are shown along with an identity bar graph and base positional differences relative to a consensus (colored bars in horizontal grey bars). [file 1471-2164-11-424-S6.PDF]
