# Supplementary material for: The Giardia lamblia vsp gene repertoire: characteristics, genomic organization, and evolution
Source: BMC Genomics. 2010 Jul 9;11:424. doi: 10.1186/1471-2164-11-424 (PMC2996952; doi:10.1186/1471-2164-11-424)
Supplement: Additional file 4 — Amino acid alignment of the 3' regions. The C-terminal 38 amino acids for all 218 complete VSPs were aligned and the alignment manually corrected. Colored shading indicates conservation of residues at particular positions across all vsps. Dashes indicate absence of a residue. At the bottom of the figure is the consensus sequence from this alignment where vertical box height correlates with frequency of occurrence of that residue. [file 1471-2164-11-424-S4.PDF]

```
                            20                            40
75    -----PRSWS GRGQRSQGAA AGF-----LV VAALAGFRRW WLLCRGKL 38
76    ---------- NSNALSPGAI TGISITVILI IGGVAGFLCW WFFCHKKK 38
2     ---------- NRSGLSPSAI AGVSVVVIIV VGGLAGFLCW WFIGRRKA 38
117   ---------T NKSGLSTGAI AGIVVAVV-I VEGLVGFLCR WFLCRSKA 38
79    ---------- TDVRLSTGAI AGISVAVIVV VGGLVGFLYW WFICRGKA 38
257   --------GA NRSGLSAGAI AGISVAVV-V VGGL-GFLCW WFVCRGKA 38
77    ---------- NKGGLSTGAI AGIAVAVIIV VGGLVGFLCW WFMCRGKA 38
88    ---------- NKSGLSAGAI AGIAVAVIIV VGGLVGFLCW WFMCRGKA 38
160   ---------- NKSGLSAGAI AGISVAVIIV VGGLVGFLCW WFMCRGKA 38
186   ---------- NKSGLSAGAI AGISVAVIIV VGGLVGFLCW WFMCRGKA 38
122   ---------- NKGGLSTGAI AGITVAVVVV VGGLAGFLCW WFICRGKA 38
52    ---------- NKSGLSTGAI AGISVTAVVV VAGLVSFLCW WFLCRGKA 38
91    ---------- NKSGLSTGAI AGIAVAAVVV VGGLVGFLCW WFIGRRKA 38
113   ---------- KSNGLSTGAI AGIVVAAVIV IGGLVGFLCW WFLCRGKA 38
3     ---------- KSGGLSTGAI AGIAVAAIIV VGGLVGFLCW WFLCRGKA 38
4     ---------- KSSGLSTGAI AGIAVAAVIV VGGLVGFLCW WFLCRGKA 38
13    ---------- KSSGLSTGAI AGIAVAAVIV VGGLVGFLCW WFLCRGKA 38
15    ---------- KSSGLSTGAI AGIAVAAVIV VGGLVGFLCW WFLCRGKA 38
23    ---------- KSSGLSTGAI AGIAVAAVIV VGGLVGFLCW WFLCRGKA 38
34    ---------- KSSGLSTGAI AGIAVAAVIV VGGLVGFLCW WFLCRGKA 38
46    ---------- KSSGLSTGAI AGIAVAAVIV VGGLVGFLCW WFLCRGKA 38
97    ---------- KSSGLSTGAI AGIAVAAVIV VGGLVGFLCW WFLCRGKA 38
128   ---------- KSSGLSTGAI AGIAVAAVIV VGGLVGFLCW WFLCRGKA 38
266   ---------- KSSGLSTGAI AGIAVAAVIV VGGLVGFLCW WFLCRGKA 38
156   ---------- KSSGLSTGAI AGIAVAAVIV VGGLVGFLCW WFICRGKA 38
18    ---------- KSSRLSTGAI AGISVAVVVV VGGLVGFLCW WFLCRGKA 38
21    ---------- KSSGLSTGAI AGISVAVVVV VGGLVGFLCW WFLCRGKA 38
90    ---------- KSSGLSTGAI AGISVAVVVV VGGLVGFLCW WFLCRGKA 38
95    ---------- KSSGLSTGAI AGISVAVVVV VGGLVGFLCW WFLCRGKA 38
9     ---------- NKSGLSTGAI AGIAVAVVIV VGGLVGFLCW WFLCRGKA 38
165   ---------- NKSGLSTGAI AGIAVAAVII VGGLVGFLCW WFLCRGKA 38
166   ---------- NKSGLSTGAI AGIAVAAVII VGGLVGFLCW WFLCRGKA 38
74    ---------- NKSGLSTGAI AGIAVAAIIV VGGLVGFLCW WFICRGKA 38
1.1   ---------- NKSGLSTGAI AGIAVAAVIV VGGLVGFLCW WFICRGKA 38
71    ---------- NKSGLSTGAI AGIAVAAVIV VGGLVGFLCW WFICRGKA 38
32    ---------- NKSGLSTGAI AGIAVAAVIV VGGLVGFLCW WFLCRGKA 38
36.1  ---------- NKSGLSTGAI AGIAVAAVIV VGGLVGFLCW WFLCRGKA 38
47.1  ---------- NKSGLSTGAI AGIAVAAVIV VGGLVGFLCW WFLCRGKA 38
51    ---------- NKSGLSTGAI AGIAVAAVIV VGGLVGFLCW WFLCRGKA 38
132   ---------- KSSGLSTGAI AGISVAVVVV VAGLVGFLCW WFICRGKA 38
8     ---------- NKSGLSAGAI AGISVAVIVV VAGLVGFLCW WFICRGKA 38
45    ---------- NKSGLSAGAI AGISVAVIVV VAGLVGFLCW WFICRGKA 38
30    ---------- NRSGLSADAI AGISVAVVVV VGGLVGFLCW WFVCRGKA 38
223   ---------- GNSGLSTGAI AGISVAVIAV VGGLVGFLCW WFVCRGKA 38
173   ---------- NRSGLSTGAI VGIFVAVIVV VGGLVGFLCW WFLCRGKA 38
251   ---------- NRSGLRTGAI AGIAVAVVAV VGGLVGFLCW WFVCRGKA 38
284   ---------- NRSGLRTGAI AGIAVAVVAV VGGLVGFLCW WFVCRGKA 38
227   ---------- NRSGLSTGAI AGIAVAVVAV VGGLVGFLCW WFLCRGKA 38
135   ---------- NRSGLSTGAI AGIAVAVVAV VGGLVGFLCW WFLCRGKA 38
213   ---------- NRSGLRTGAI AGIAVAVVAV VGGLVGFLCW WFLCRGKA 38
214   ---------- NRSGLRTGAI AGIAVAVVAV VGGLVGFLCW WFLCRGKA 38
212   ---------S NRSGL-TGAI AGIAVAVVAV VGGLVGFLCW WFLCRGKA 38
254   --------GT NRSGLSTGAI AGISVAVIAV VGGLVGFLCW WF-C-GKA 38
78    ---------- NKSGLSTGAI AGISVAAVVV VGGLVGFLCW WFVCRGKA 38
145   ---------- NKSGLSTGAI AGISVAVVVV VGGLVGFLCW WFVCRGKA 38
31    ---------- NKSGLSTGAI AGISVAVVAV VGGLVGFLCW WFLCRGKA 38
204   ---------- NKSGLSTGAI AGISVAVVAV VGGLVGFLCW WFLCRGKA 38
112   ---------- NKSGLSTGAI AGISVAVIAV VGGLVGFLCW WFLCRGKA 38
49    ---------- NKSGLSTGTI AGISVAVIVV VGGLVGFLCW WFVCRGKA 38
25    ---------- NKSGLSTGAI AGISVAVIVV VGGLVGFLCW WFVCRGKA 38
26.1  ---------- NKSGLSTGAI AGISVAVIVV VGGLVGFLCW WFVCRGKA 38
127   ---------- NKSGLSTGAI AGISVAVIVV VGGLVGFLCW WFVCRGKA 38
121   ---------- NKSGLSTGAI AGISVAVIAV VAGLVGFLCW WFVCRGKA 38
109   ---------- NKSGLSTGAI AGISVAVIAV VGGLVGFLCW WFVCRGKA 38
238   ---------- NKSGLSTGAI AGISVAVIAV VGGLVGFLCW WFVCRGKA 38
249   ---------- NKSGLSTGAI AGISVAVIAV VGGLVGFLCW WFVCRGKA 38
265   ---------- NKSGLSTGAI AGISVAVIAV VGGLVGFLCW WFVCRGKA 38
285   ---------- NKSGLSTGAI AGISVAVIAV VGGLVGFLCW WFVCRGKA 38
84    ---------- NRSGLSTGAV AGISVAVIAV VGGLVGFLCW WFVCRGKA 38
276   ---------- NRSGLSTGAI AGISVAVIAV VGGLVGFLCW WFVWRGKA 38
83    ---------- NRSGLSTGAI AGISVAVIAV VGGLVGFLCW WFVCRGKA 38
85    ---------- NRSGLSTGAI AGISVAVIAV VGGLVGFLCW WFVCRGKA 38
94    ---------- NRSGLSTGAI AGISVAVIAV VGGLVGFLCW WFVCRGKA 38
102   ---------- NRSGLSTGAI AGISVAVIAV VGGLVGFLCW WFVCRGKA 38
111   ---------- NRSGLSTGAI AGISVAVIAV VGGLVGFLCW WFVCRGKA 38
137   ---------- NRSGLSTGAI AGISVAVIAV VGGLVGFLCW WFVCRGKA 38
191   ---------- NRSGLSTGAI AGISVAVIAV VGGLVGFLCW WFVCRGKA 38
193   ---------- NRSGLSTGAI AGISVAVIAV VGGLVGFLCW WFVCRGKA 38
216   ---------- NRSGLSTGAI AGISVAVIAV VGGLVGFLCW WFVCRGKA 38
222   ---------- NRSGLSTGAI AGISVAVIAV VGGLVGFLCW WFVCRGKA 38
228   ---------- NRSGLSTGAI AGISVAVIAV VGGLVGFLCW WFVCRGKA 38
234   ---------- NRSGLSTGAI AGISVAVIAV VGGLVGFLCW WFVCRGKA 38
235   ---------- NRSGLSTGAI AGISVAVIAV VGGLVGFLCW WFVCRGKA 38
246   ---------- NRSGLSTGAI AGISVAVIAV VGGLVGFLCW WFVCRGKA 38
248   ---------- NRSGLSTGAI AGISVAVIAV VGGLVGFLCW WFVCRGKA 38
```

255	----------	NRSGLSTGAI	AGISVAVIAV	VGGLVGFLCW	WFVCRGKA	38
268	----------	NRSGLSTGAI	AGISVAVIAV	VGGLVGFLCW	WFVCRGKA	38
271	----------	NRSGLSTGAI	AGISVAVIAV	VGGLVGFLCW	WFVCRGKA	38
272	----------	NRSGLSTGAI	AGISVAVIAV	VGGLVGFLCW	WFVCRGKA	38
279	----------	NRSGLSTGAI	AGISVAVIAV	VGGLVGFLCW	WFVCRGKA	38
17	----------	NKSELSTGAI	AGIAVAVIVV	VGGLVGFLCW	WFLCRGKA	38
130	----------	NKSGLSTGAI	AGIAVAVIVV	VGGLVGFLCW	WFLCRGKA	38
188	----------	NKSGLSTGAI	AGIAVAVIVV	VGGLVGFLCW	WFLCRGKA	38
37	----------	NKSGLSTGAI	AGIAVAVIVI	VGGLVGFLCW	WFLCRGKA	38
118	----------	NKSGLSTGAI	AGISVAVIVI	VGGLVGFLCW	WFLCRGKA	38
5	----------	NKSGLSTGAI	AGISVAVIVV	VGGLIGFLCW	WFLCRGKA	38
172	----------	NKSGLSTGAI	AGISVAVIVV	VGGLVGFLCW	WFLYRGKA	38
115	----------	NKSGLSMGAI	AGISVAVIVV	VGGLVGFLCW	WFLCRGKA	38
6	----------	NKSGLSTGAI	AGISVAVIVV	VGGLVGFLCW	WFLCRGKA	38
16	----------	NKSGLSTGAI	AGISVAVIVV	VGGLVGFLCW	WFLCRGKA	38
24	----------	NKSGLSTGAI	AGISVAVIVV	VGGLVGFLCW	WFLCRGKA	38
27	----------	NKSGLSTGAI	AGISVAVIVV	VGGLVGFLCW	WFLCRGKA	38
89	----------	NKSGLSTGAI	AGISVAVIVV	VGGLVGFLCW	WFLCRGKA	38
100	----------	NKSGLSTGAI	AGISVAVIVV	VGGLVGFLCW	WFLCRGKA	38
101	----------	NKSGLSTGAI	AGISVAVIVV	VGGLVGFLCW	WFLCRGKA	38
108	----------	NKSGLSTGAI	AGISVAVIVV	VGGLVGFLCW	WFLCRGKA	38
161	----------	NKSGLSTGAI	AGISVAVIVV	VGGLVGFLCW	WFLCRGKA	38
187	----------	NKSGLSTGAI	AGISVAVIVV	VGGLVGFLCW	WFLCRGKA	38
189	----------	NKSGLSTGAI	AGISVAVIVV	VGGLVGFLCW	WFLCRGKA	38
236	----------	NKSGLSTGAI	AGISVAVIVV	VGGLVGFLCW	WFLCRGKA	38
275	----------	NKSGLSTGAI	AGISVAVIVV	VGGLVGFLCW	WFLCRGKA	38
159	----------	NKSGLSTGAI	AGISVAAIVV	VGGLVGFLCW	WFICRGKA	38
181	----------	NKSGLSTGAI	AGISVAAIVV	VGGLVGFLCW	WFICRGKA	38
182	----------	NKSGLSTGAI	AGISVAAIVV	VGGLVGFLCW	WFICRGKA	38
185	----------	NKSGLSTGAI	AGISVAAIVV	VGGLVGFLCW	WFICRGKA	38
180	----------	NKSGLSTGAI	AGISVAAIVV	VGGLIGFLCW	WFICRGKA	38
19	----------	NRSGLSTGAI	AGISVAVIVV	VGGLIGFLCW	WFICRGKA	38
20.1	----------	NKSGLSTGAI	AGISVAVIVV	VGGLIGFLCW	WFICRGKA	38
28	----------	NKSGLSTGAI	AGISVAVIVV	VGGLIGFLCW	WFICRGKA	38
126.1	----------	NKSGLSTGAI	AGISVAVIVV	VGGLIGFLCW	WFICRGKA	38
176	----------	NRSGLSTGAI	AGISVAVIVV	IGGLVGFLCW	WFVCRGKA	38
177	----------	NRSGLSTGAI	AGISVAVIVV	IGGLVGFLCW	WFVCRGKA	38
178	----------	NRSGLSTGAI	AGISVAVIVV	IGGLVGFLCW	WFVCRGKA	38
179	----------	NRSGLSTGAI	AGISVAVIVV	IGGLVGFLCW	WFVCRGKA	38
241	----------	NRSGLSTGAI	AGISVAVIVV	IGGLVGFLCW	WFVCRGKA	38
242	----------	NRSGLSTGAI	AGISVAVIVV	IGGLVGFLCW	WFVCRGKA	38
129	----------	NKSGLSTGAI	AGISVAVIVI	VGGLVGFLCW	WFICRGKA	38
107.1	----------	NRSGLSTGAI	AGISVAVIVV	VGGLIGFLCW	WFVCRGKA	38
70	----------	NRSGLSTGAI	AGISVAVIVV	VGGLVGFLCW	WFLCRGKA	38
86	----------	NRSGLSTGAI	AGISVAVIVV	VGGLVGFLCW	WFLCRGKA	38
29	----------	NKSGLSTGAI	AGISVAVIVV	VGGLVGFLCW	WFICRGKA	38
33	----------	NKSGLSTGAI	AGISVAVIVV	VGGLVGFLCW	WFICRGKA	38
35	----------	NKSGLSTGAI	AGISVAVIVV	VGGLVGFLCW	WFICRGKA	38
41	----------	NKSGLSTGAI	AGISVAVIVV	VGGLVGFLCW	WFICRGKA	38
42	----------	NKSGLSTGAI	AGISVAVIVV	VGGLVGFLCW	WFICRGKA	38
53.1	----------	NKSGLSTGAI	AGISVAVIVV	VGGLVGFLCW	WFICRGKA	38
87.1	----------	NKSGLSTGAI	AGISVAVIVV	VGGLVGFLCW	WFICRGKA	38
114	----------	NKSGLSTGAI	AGISVAVIVV	VGGLVGFLCW	WFICRGKA	38
116	----------	NKSGLSTGAI	AGISVAVIVV	VGGLVGFLCW	WFICRGKA	38
141	----------	NKSGLSTGAI	AGISVAVIVV	VGGLVGFLCW	WFICRGKA	38
162.1	----------	NKSGLSTGAI	AGISVAVIVV	VGGLVGFLCW	WFICRGKA	38
167	----------	NKSGLSTGAI	AGISVAVIVV	VGGLVGFLCW	WFICRGKA	38
168.1	----------	NKSGLSTGAI	AGISVAVIVV	VGGLVGFLCW	WFICRGKA	38
183	----------	NKSGLSTGAI	AGISVAVIAV	VGGLVGFLCW	WFICRGKA	38
195	----------	NKSGLSTGAI	AGISVAVIAV	VGGLVGFLCW	WFICRGKA	38
231	----------	NKSGLSTGAI	AGISVAVIAV	VGGLVGFLCW	WFICRGKA	38
92	----------	NRSGLSTGAI	AGISVAVIAV	VGGLVGFLCW	WFICRGKA	38
103	----------	NRSGLSTGAI	AGISVAVIAV	VGGLVGFLCW	WFICRGKA	38
110	----------	NRSGLSTGAI	AGISVAVIAV	VGGLVGFLCW	WFICRGKA	38
61	----------	NRSGLSTGAI	AGISVAVIAV	VGGLIGFLCW	WFVCRGKA	38
63	----------	NRSGLSTGAI	AGISVAVIAV	VGGLIGFLCW	WFVCRGKA	38
14	----------	NRSGLSTGAI	AGISVAVIAV	VGGLVGFLCW	WFLCRGKA	38
64	----------	NRSGLSTGAI	AGISVAVIAV	VGGLVGFLCW	WFLCRGKA	38
106	----------	NRSGLSTGAI	AGISVAVIAV	VGGLVGFLCW	WFLCRGKA	38
123	----------	NRSGLSTGAI	AGISVAVIAV	VGGLVGFLCW	WFLCRGKA	38
124	----------	NRSGLSTGAI	AGISVAVIAV	VGGLVGFLCW	WFLCRGKA	38
65	----------	NRSGLSTGAI	AGISVAVIAV	VGGLIGFLCW	WFLCRGKA	38
10	----------	NRGGLSTGAI	AGISVAVIVV	VGGLVGFLCW	WFICRGKA	38
59	----------	NRGGLSTGAI	AGISVAVIVV	VGGLVGFLCW	WFICRGKA	38
174	----------	NRSGLSTGAI	AGISVAVIVV	VAGLVGFLCW	WFICRGKA	38
38	---------T	NRSGLSTGAI	AGISVAVI-V	VGGLVGFLCW	WFICRGKA	38
43	----------	NRSGLSTGAI	AGISVAVIVV	VGGLVGFLCW	WFICRGKA	38
171	----------	NRSGLSTGAI	AGISVAVIVV	VGGLVGFLCW	WFICRGKA	38
99	AGTSCVSMST	NRGGLSTGAI	AGIS-----V	VAGLVGFL--	---CRGKA	38
286	----------	NRSALSTGAI	AGISVAVVAV	VGGLVGFLCW	WFICRGKA	38
175	----------	NKSGLSTGAI	AGIAVAVVVV	VGGLVGFLCW	WFICRGKA	38
225	---------V	NKSGLSTGAI	A-ISVAVVVV	VGGLVGFLCW	WFICRGKA	38
7.1	----------	NKSGLSTGAI	AGISVAVVVV	VGGLVGFLCW	WFICRGKA	38
48.1	----------	NKSGLSTGAI	AGISVAVVVV	VGGLVGFLCW	WFICRGKA	38
66	----------	NKSGLSTGAI	AGISVAVVVV	VGGLVGFLCW	WFICRGKA	38
67	----------	NKSGLSTGAI	AGISVAVVVV	VGGLVGFLCW	WFICRGKA	38

```
131  ----------  SRSGLSTGAI  AGISVAVVVV  VAGLVGFLCW  WFICRGKA  38
184  ----------  NKSGLSTGAI  AGISVAVVVV  VAGLVGFLCW  WFICRGKA  38
72   ----------  NRSGLSTGAI  AGISVAVVVV  VAGLVGFLCW  WFICRGKA  38
73   ----------  NRSGLSTGAI  AGISVAVVVV  VAGLVGFLCW  WFICRGKA  38
169  ----------  NKSGLSTGAI  AGISVAAVVI  VAGLVGFLCW  WFICRGKA  38
142  ----------  NRSGLSTGAI  AGISVAAVVV  VAGLVGFLCW  WFICRGKA  38
98.1 ----------  NKSGLSTGAI  AGISVAAFVV  VAGLVGFLCW  WFICRGKA  38
54   ----------  NKSGLSTGAI  AGISVAAVVV  VAGLVGFLCW  WFICRGKA  38
170  ----------  NRGGLSTGAI  AGISVAVVVV  VGGLVGFLCW  WFLCRGKA  38
81   ----------  NRSGLSAGAI  AGISVAVVAV  VGGLVGFLCW  WFVCRGKA  38
119  ----------  NRSGLSAGAI  AGISVAVVAV  VGGLVGFLCW  WFVCRGKA  38
138  ----------  NRSGLSAGAI  AGISVAVVAV  VGGLVGFLCW  WFVCRGKA  38
139  ----------  NRSGLSAGAI  AGISVAVVAV  VGGLVGFLCW  WFVCRGKA  38
158  ----------  NRSGLSAGAI  AGISVAVVAV  VGGLVGFLCW  WFVCRGKA  38
217  ----------  NRSGLSAGAI  AGISVAVVAV  VGGLVGFLCW  WFVCRGKA  38
267  ----------  NRSGLSAGAI  AGISVAVVAV  VGGLVGFLCW  WFVCRGKA  38
11   ----------  NRGGLSTGAI  AGISVAVVAV  VGGLVGFLCW  WFLCRGKA  38
290  ----------  NRSGLSTGAI  AGISVAVVAV  VGGLVGFLCW  WFVCRGKA  38
277  ----------  NRSGLSTGAI  AGISVAVVAV  VGGLVGFLCW  WFVCRGKA  38
270  ----------  NRSGLSTGAI  AGISVAVVAV  VGGLVGFLCW  WFVCRGKA  38
261  ----------  NRSGLSTGAI  AGISVAVVAV  VGGLVGFLCW  WFVCRGKA  38
233  ----------  NRSGLSTGAI  AGISVAVVAV  VGGLVGFLCW  WFVCRGKA  38
220  ----------  NRSGLSTGAI  AGISVAVVAV  VGGLVGFLCW  WFVCRGKA  38
218  ----------  NRSGLSTGAI  AGISVAVVAV  VGGLVGFLCW  WFVCRGKA  38
196  ----------  NRSGLSTGAI  AGISVAVVAV  VGGLVGFLCW  WFVCRGKA  38
192  ----------  NRSGLSTGAI  AGISVAVVAV  VGGLVGFLCW  WFVCRGKA  38
136  ----------  NRSGLSTGAI  AGISVAVVAV  VGGLVGFLCW  WFVCRGKA  38
80   ----------  NRSGLSTGAI  AGISVAVVAV  VGGLVGFLCW  WFVCRGKA  38
62   ----------  NRSGLSTGAI  AGISVAVVAV  VGGLVGFLCW  WFVCRGKA  38
60   ----------  NRSGLSTGAI  AGISVAVVAV  VGGLVGFLCW  WFVCRGKA  38
39   -----VSSGT  NRSGLSTGAI  AGISVAVVAV  VGGLVGFL--  ---CRGKA  38
44   ----------  NRSGLSTGAI  AGISVAVVAV  VGGLVGFLCW  WFLCRGKA  38
68   ----------  NRSGLSTGAI  AGISVAVVAV  VGGLVGFLCW  WFLCRGKA  38
262  ----------  NRSGLSTGAI  AGISVAVVAV  VGGLVGFLCW  WFLCRGKA  38
134  ----------  NRGGLSTGAI  AGISVAVVVV  VGGLIGFLCW  WFICRGKA  38
163  ----------  NRGGLSTGAI  AGISVAVVVV  VGGLIGFLCW  WFICRGKA  38
289  ----------  NRGGLSTGAI  AGISVAVVVV  VGGLIGFLCW  WFICRGKA  38
215  ----------  NRGGLSTGAI  AGISVAVVVV  VGGLIGFLCW  WFVCRGKA  38
57   ----------  NRSGLSAGAI  AGIAVAVVVV  VGGLVGFLCW  WFICRGKA  38
287  ----------  NRSGLSAGAI  AGIAVAVVVV  VGGLVGFLCW  WFICRGKA  38
190  ----------  NRSGLSTGAI  AGIAVAVVVV  VGGLVGFLCW  WFICRGKA  38
201  ----------  NRSGLSTGAI  AGIAVAVVVV  VGGLVGFLCW  WFICRGKA  38
209  ----------  NRSGLSTGAI  AGIAVAVVVV  VGGLVGFLCW  WFICRGKA  38
12   ----------  NRGGLSTGAI  AGISVAVVVV  VGGLVGFLCW  WFVCRGKA  38
105  ----------  NRGGLSTGAI  AGISVAVVVV  VGGLVGFLCW  WFVCRGKA  38
143  ----------  NRGGLSTGAI  AGISVAVVVV  VGGLVGFLCW  WFVCRGKA  38
40   ----------  NRGGLSTGAI  AGISIAVVVV  VGGLVGFLCW  WFVCRGKA  38
144  ----------  NRSGLSTGAV  AGISVAVVVV  VGGLVGFLCW  WFVCRGKA  38
203  ----------  NRSGLSTGAV  AGISVAVVVV  VGGLVGFLCW  WFVCRGKA  38
247  ----------  NRSGLSTGAV  AGISVAVVVV  VGGLVGFLCW  WFVCRGKA  38
82   ----------  NRSGLSTGAI  AGISVAVVVV  VGGLVGFLCW  WFICRGKA  38
120  ----------  NRSGLSTGAI  AGISVAVVVV  VGGLVGFLCW  WFICRGKA  38
140  ----------  NRSGLSTGAI  AGISVAVVVV  VGGLVGFLCW  WFICRGKA  38
149  ----------  NRSGLSTGAI  AGISVAVVVV  VGGLVGFLCW  WFICRGKA  38
150  ----------  NRSGLSTGAI  AGISVAVVVV  VGGLVGFLCW  WFICRGKA  38
151  ----------  NRSGLSTGAI  AGISVAVVVV  VGGLVGFLCW  WFICRGKA  38
153  ----------  NRSGLSTGAI  AGISVAVVVV  VGGLVGFLCW  WFICRGKA  38
157  ----------  NRSGLSTGAI  AGISVAVVVV  VGGLVGFLCW  WFICRGKA  38
199  ----------  NRSGLSTGAI  AGISVAVVVV  VGGLVGFLCW  WFICRGKA  38
200  ----------  NRSGLSTGAI  AGISVAVVVV  VGGLVGFLCW  WFICRGKA  38
202  ----------  NRSGLSTGAI  AGISVAVVVV  VGGLVGFLCW  WFICRGKA  38
206  ----------  NRSGLSTGAI  AGISVAVVVV  VGGLVGFLCW  WFICRGKA  38
210  ----------  NRSGLSTGAI  AGISVAVVVV  VGGLVGFLCW  WFICRGKA  38
221  ----------  NRSGLSTGAI  AGISVAVVVV  VGGLVGFLCW  WFICRGKA  38
224  ----------  NRSGLSTGAI  AGISVAVVVV  VGGLVGFLCW  WFICRGKA  38
243  ----------  NRSGLSTGAI  AGISVAVVVV  VGGLVGFLCW  WFICRGKA  38
245  ----------  NRSGLSTGAI  AGISVAVVVV  VGGLVGFLCW  WFICRGKA  38
259  ----------  NRSGLSTGAI  AGISVAVVVV  VGGLVGFLCW  WFICRGKA  38
263  ----------  NRSGLSTGAI  AGISVAVVVV  VGGLVGFLCW  WFICRGKA  38
69   ----------  NRSGLSTGAI  AGISVAVVVV  VGGLVGFLCW  WFLCRGKA  38
152  ----------  NRSGLSTGAI  AGISVAVVVV  VGGLVGFLCW  WFLCRGKA  38
154  ----------  NRSGLSTGAI  AGISVAVVVV  VGGLVGFLCW  WFLCRGKA  38
50   ----------  NRSGLSTGAI  AGISVAVVVV  VGGLIGFLCW  WFVCRGKA  38
56   ----------  NRSGLSTGAI  AGISVAVVVV  VGGLVGFLCW  WFVCRGKA  38
58   ----------  NRSGLSTGAI  AGISVAVVVV  VGGLVGFLCW  WFVCRGKA  38
93   ----------  NRSGLSTGAI  AGISVAVVVV  VGGLVGFLCW  WFVCRGKA  38
104  ----------  NRSGLSTGAI  AGISVAVVVV  VGGLVGFLCW  WFVCRGKA  38
146  ----------  NRSGLSTGAI  AGISVAVVVV  VGGLVGFLCW  WFVCRGKA  38
147  ----------  NRSGLSTGAI  AGISVAVVVV  VGGLVGFLCW  WFVCRGKA  38
198  ----------  NRSGLSTGAI  AGISVAVVVV  VGGLVGFLCW  WFVCRGKA  38
207  ----------  NRSGLSTGAI  AGISVAVVVV  VGGLVGFLCW  WFVCRGKA  38
240  ----------  NRSGLSTGAI  AGISVAVVVV  VGGLVGFLCW  WFVCRGKA  38
250  ----------  NRSGLSTGAI  AGISVAVVVV  VGGLVGFLCW  WFVCRGKA  38
```

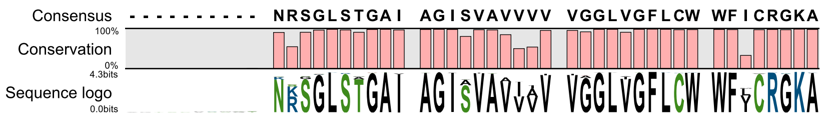
Consensus
- - - - - - - - - - NRSGLSTGAI AGISVAVVVV VGGLVGFLCW WFICRGKA
Conservation
100%
0%
Sequence logo
4.3bits
0.0bits
